# Intimidation against advocates and researchers in the tobacco, alcohol and ultra-processed food spaces: a review

**DOI:** 10.1093/heapro/daae153

**Published:** 2024-11-21

**Authors:** Karen A Evans-Reeves, Britta K Matthes, Phil Chamberlain, Nino Paichadze, Anna B Gilmore, Melissa Mialon

**Affiliations:** Tobacco Control Research Group, Department for Health, University of Bath, The Avenue, Claverton Down, Bath, Bath and North East Somerset, BA2 7AY, UK; Tobacco Control Research Group, Department for Health, University of Bath, The Avenue, Claverton Down, Bath, Bath and North East Somerset, BA2 7AY, UK; Tobacco Control Research Group, Department for Health, University of Bath, The Avenue, Claverton Down, Bath, Bath and North East Somerset, BA2 7AY, UK; Department of Global Health and the Center on Commercial Determinants of Health, Milken Institute School of Public Health, The George Washington University, 950 New Hampshire Avenue NW, Washington, DC 20052, USA; Tobacco Control Research Group, Department for Health, University of Bath, The Avenue, Claverton Down, Bath, Bath and North East Somerset, BA2 7AY, UK; Universite Rennes, EHESP, CNRS, Inserm, Arènes—UMR 6051, RSMS (Recherche sur les Services et Management en Santé)—U 1309, Rennes, France

**Keywords:** intimidation, surveillance, legal challenges, public discreditation, corporate political activity

## Abstract

Unhealthy commodity industries (UCIs) engage in corporate political activity, using diverse practices, including intimidatory tactics, to thwart, delay and dilute regulations that threaten their businesses. While examples of such intimidation exist across multiple sectors, no attempt has been made to synthesize these. Furthermore, much of the literature focuses on intimidation of policy-makers. Less is known about the types of intimidation experienced by advocates and researchers and their responses to this intimidation. This scoping review explores the literature across the tobacco, alcohol and ultra-processed food spaces for instances of intimidation and categorizes them inductively and deductively based on a framework of intimidation types. Similarly, responses to intimidation were mapped onto a pre-existing framework. We found intimidatory tactics towards advocates and researchers in every sector. Public discreditation, followed by legal threats and action, complaints and freedom of information requests were most frequently mentioned and often attributed to UCIs or their third parties. Surveillance, threats of violence, violence, burglary and bribery were less prevalent in the literature and their perpetrators were unknown. Those intimidated reported carrying on as normal, defensive action (changing/adapting work, taking security precautions) or, as was most reported, offensive action (exposing intimidation, correcting misinformation, taking legal action). The similarity of intimidation across sectors suggests that UCIs engage in similar intimidatory tactics regardless of sector. Understanding more about the scale of intimidation and how it impacts the work and wellbeing of those affected is essential, as is learning more about the ways researchers and advocates can effectively pre-empt and respond.

**Contribution to Health Promotion**

- Exposes the types of intimidation experienced by advocates and researchers working in tobacco, alcohol and ultra-processed food control as well as the impacts of this intimidation.
- Exposing intimidation may weaken its impact, namely, the chilling effect it has on work which aims to secure the health of nations.
- Highlights the opportunities for collaboration across sectors to develop a public health community of support which could lead to effective counter strategies against corporate intimidation.

## INTRODUCTION

Non-communicable diseases now account for nearly three-quarters of all deaths globally. Policy-makers are increasingly recognizing the need to regulate the vectors of these diseases, the unhealthy commodity industries (UCIs) (Gilmore *et al*., 2023; WHO, 2023). Tobacco, alcohol and ultra-processed food (UPF; including the sugar-sweetened beverage [SSB] and infant formula) industries have used corporate political activity (CPA) to delay or prevent regulations that threaten their businesses. While most research has focused on corporate influence on governments and policy-makers, research has also revealed UCI attempts at monitoring, undermining and discrediting, those who campaign and provide evidence for increased regulation (Bialous *et al*., 2001; Ibrahim and Glantz, 2007; Vedwan, 2007; Brownell and Warner, 2009; Landman and Glantz, 2009; Moodie *et al*., 2013; Avery *et al*., 2016; Savell *et al*., 2016; Ulucanlar *et al*., 2016, 2023; Matthes *et al*., 2021; Reed *et al*., 2021; Lauber *et al*., 2022). However, the scale of the intimidation experienced by advocates and researchers, its form and impact, as well as the responses of those intimidated, have not been examined in detail across UCI sectors. There is a growing appetite for cross-sector research to identify common CPA strategies used by UCIs to help policy-makers act in favour of health (Maani *et al*., 2020; Bhuptani *et al*., 2022).

Documents from Philip Morris International (PMI) made public after litigation against the company in the late 1990s showed that it felt it was in ‘a state of war’ with the ‘anti-tobacco brigade’, which was ‘marching ever forward’ (Knight and Chapman, 2004). To try and stop tobacco control progress, PMI developed both the ‘Long Range Plan’ (Ulucanlar *et al*., 2016) and the ‘Fair Play Strategy’ (McDaniel *et al*., 2006), which outlined four main strategies to undermine tobacco control advocates and researchers. First, it planned to intensify research on the tobacco control community; second, it would build relationships with ‘moderate’ tobacco control organizations; third, it planned to diminish tobacco control funding; and fourth, it planned to weaken the credibility of tobacco control organizations and their leadership (McDaniel *et al*., 2006; Slavitt, 1997). In the decades that followed, those working in tobacco control experienced reputational attacks in the media, online harassment, legal threats and action, threats of death and violence, freedom of information (FOI) requests, surveillance and theft (Matthes *et al*., 2023).

Advocates and researchers working on UPF (including SSB and infant formula) (Chatterjee, 2013; Jacobs and Richtel, 2017; Lorenzo, 2019; Lauber *et al*., 2021; Mialon, 2021) and alcohol (Hager, 2014; Matanje Mwagomba *et al*., 2018) have been targeted with the same intimidatory tactics as those working in tobacco control, but these are less thoroughly documented. This is likely due to the legacy library cache of previously secret internal tobacco industry documents, which enabled researchers to triangulate the tobacco industry’s explicit plans with advocates’ and academics’ reported experiences.

Given the similarities in CPA between UCIs more broadly, there is a need to examine intimidation across UCIs, an issue not hitherto addressed in the literature. This scoping review aims to examine and categorize the types of intimidation experienced by advocates and researchers working in the tobacco, alcohol and UPF sectors and the responses by these individuals to the intimidation they have experienced. The results may help those working in different sectors to learn from each other, both in terms of what types of intimidation to expect and how to respond.

## METHODS

We conducted a scoping review of the literature as this area of research remains underdeveloped (Colquhoun *et al*., 2014). We used the Preferred Reporting Items for Systematic Reviews and Meta-Analyses’ Extension for Scoping Reviews (PRIMA-ScR) when preparing, conducting and writing up this scoping review (Tricco *et al*., 2018).

### Study identification and selection

#### Search strategy

We used the following search terms: (‘alcohol industry’ OR ‘beverage industry’ OR ‘drink industry’ OR ‘food industry’ OR ‘tobacco industry’) AND (‘attack*’ OR ‘intimidat*’ OR ‘threat*’ OR ‘interfere*’) AND (‘advocat*’ OR ‘researcher*’). These terms were based on the collective knowledge of the research team, and existing work on the topic.

We searched six databases: Embase, International Bibliography of the Social Sciences (IBSS), JSTOR, PubMed, Scopus, Web of Science, including health (e.g. PubMed) and social science databases (e.g. IBSS) to ensure relevant literature from various fields was identified.

Searches were conducted in February 2022 and included literature published between 01/2000 and 02/2022. To ensure that we uncovered contemporaneous instances of industry tactics and to limit the volume of potential papers, we chose 2000 as a starting date. It was in this decade that the World Health Organization (WHO) recognized that, in addition to tobacco use, unhealthy diets and harmful alcohol drinking were major health issues, and thus developed global action plans for tobacco, food and alcohol products which subsequently threatened these sectors with regulation and prompted counter action from UCIs (Lauber *et al*., 2021; Rinaldi *et al*., 2022).

#### Inclusion and exclusion criteria

To be included, an article had to draw on primary data and/or policy or other documents and report one or more instances of intimidation—understood broadly following (Matthes *et al*., 2022) as ‘*actions that make[s] [one] feel frightened or threatened*’(Matthes *et al*., 2022)—of advocates or researchers working on public health in the broad areas of tobacco, alcohol or UPF. We excluded sources (i) where the target of the intimidation was not an advocate, a researcher or an advocacy or research organization, (ii) the mention of intimidation was too vague to categorize meaningfully, (iii) the search terms were included in the article, but the content was irrelevant, (iv) sources not published in English, French, German or Spanish (languages spoken by our research team) and (v) retracted articles (not withdrawn because of industry pressure).

#### Study selection

All search results were imported into EndNote and duplicates were removed. Next, two researchers scanned abstracts to exclude those articles that did not meet our selection criteria. We sought full texts of the remaining articles to assess these for eligibility. To ensure a common understanding of the inclusion criteria, we ran two pilots (Pilot 1: *n* = 6; Pilot 2: *n* = 20), during which three researchers (B.M., K.E.R., P.C.) independently assessed a small subset of papers and discussed their inclusion. This helped clarify the scope of intimidation, for example, also including (attempted) bribery. We then assessed the eligibility of the remaining papers. In each case, one researcher read the full text and inclusion was regularly discussed among the three researchers until a consensus was reached.

#### Additional searches

In May and June 2022, we searched for additional articles citing included papers using Google Scholar and searched the reference lists of included articles. Beyond the peer-reviewed literature, we searched the websites of 29 organizations working on UCI practices. The list of organizations was compiled based on collective knowledge of the research team. We also conducted Google and Nexis searches using relevant search terms and screened the first 100 results of each database (sorted by relevance). The purpose of these searches was not to create another systematic dataset but to explore whether there were types of intimidation covered in the grey literature (e.g. in news media) that were not covered in the peer-reviewed literature. Decisions on additional inclusions were made by consensus.

### Data charting and analysis

We extracted all incidences of intimidation into an Excel spreadsheet and recorded article metadata, relevant industry and country/region where the incidence happened, when this intimidation occurred, and extracted the relevant passage(s) from the articles. Coding consisted of three steps. First, we coded the types of intimidation described, using the typology proposed in a previous study on intimidation in tobacco control as a starting point (Matthes *et al*., 2022) but remained open to identifying further types. Where possible, we indicated who the perpetrators of the intimidation were within each of the intimidation types. Secondly, we coded the impacts of intimidation described in the included literature. Thirdly, we coded responses to intimidation, using an existing framework as a starting point (Matthes *et al*., 2023) as well as any reported outcomes of the responses. Each extract was coded independently by two of five researchers (K.E.R., B.M., P.C., N.P., M.M.), and discrepancies were discussed among all researchers until a consensus was reached.

## RESULTS

**Fig. 1:**
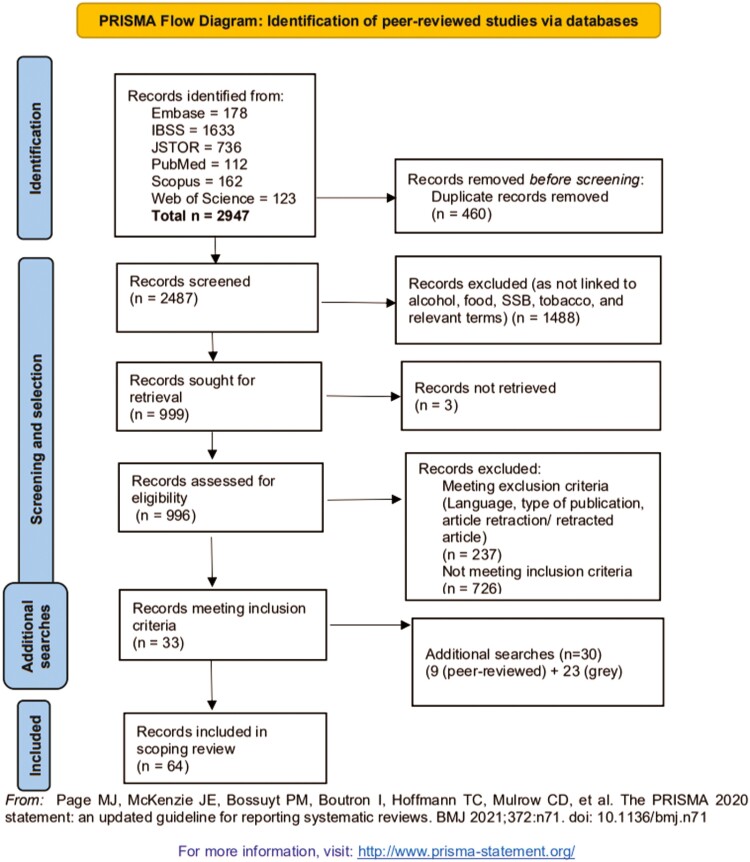
PRISMA diagram of scoping review literature selection.

Figure 1 presents the PRISMA flow diagram of this review.

All 64 included sources were published between 2000 and 2021, 12 (18.8%) between 2000 and 2005, 16 between 2006 and 2010 (25.0%), 12 (18.8%) between 2010 and 2015 and 24 (37.5%) between 2015 and 2021.

Almost two-thirds of the sources (*n* = 41, 64.1%) were peer-reviewed journal articles and the other third (*n* = 23, 35.9%) were grey literature sources, including blogs (*n* = 9, 14.1%) (CCF, 2004, 2010; Pollan, 2006; Freudenberg, 2007; Heisel, 2011; Chatterjee, 2013; Nestle, 2018; Lorenzo, 2019; TobaccoTactics, 2020), newspaper articles (*n* = 8, 12.5%) (Boseley, 2003; Campbell and Meikle, 2012; Taubes and Kearns Couzens, 2012; Huehnergarth, 2016; Jacobs and Richtel, 2017; Peres, 2017; McNeil, 2018; Espiner, 2021), news stories in peer-reviewed journals (*n* = 2, 3.1%) (Simpson, 2005; Daube, 2015), a case-study (*n* = 1, 1.6%) (RESYST, 2019), a press release (*n* = 1, 1.6%) (Foundation for Alcohol Research and Education, 2017), a recorded seminar (*n* = 1, 1.6%) (Mialon, 2021) and a book (*n* = 1, 1.6%) (Hager, 2014). For inclusiveness, we henceforth discuss the articles and other publication mediums as ‘sources’.

In the dataset, 41 sources (64.1%) discussed intimidations that occurred in high-income countries (HICs) while 18 sources (28.1%) discussed intimidations in low- and middle-income countries (LMICs), mostly located in Asia and South America, with just three sources talking about intimidation in Africa (Table 1). The remaining sources discussed intimidation at global or regional level (*n* = 5, 7.8%). Over half the sources in the dataset discussed intimidation that occurred in the USA (*n* = 23, 35.9%). Most of the US sources referred to the tobacco sector. In fact, most sources in this review covered incidents of intimidation in the tobacco sector (*n* = 40, 62.5%), followed by the UPF sector (*n* = 25, 39.1%) (the SSB sector accounted for seven of these sources) and finally the alcohol sector (*n* = 6, 9.4%; Table 2). Three sources mentioned more than one sector. Several sources discussed more than one form of intimidation, more than one instance, or more than one country.

**Table 1:** Number of articles including intimidation by country and income

Country/Region	Peer	References	Grey literature	References	Total
Low income					
Malawi	1	Matanje Mwagoma *et al*, 2018			1
Low-middle income					
India	2	Vedwan, 2007; Goel *et al*., 2021			2
Indonesia			1	**McNeill, 2018**	1
Nepal	2	Bhatta *et al.*, 2020a, b			2
Nigeria	1	Egbe *et al*., 2019	1	**McNeill, 2018**	2
Philippines	1	Baker *et al*., 2021			1
Ukraine	1	Hoe *et al*., 2021			1
‘Islamic countries’^a^	1	Petticrew *et al*., 2015			1
Upper-middle income					
Argentina	1	Mejia *et al*., 2008			1
Brazil			2	Peres, 2017; **Mialon, 2021**	2
Colombia			1	**Jacobs and Richtel, 2017**	1
Fiji			1	**Mialon, 2021**	1
Mexico			1	**Jacobs, 2017**	1
South Africa			1	RESYST, 2019	1
Thailand	3	Charoenca *et al.*, 2012; Assunta *et al*., 2017; Patanavanich and Glantz, 2021			3
High income					
Australia	1	Avery *et al*., 2016	3	**Mialon, 2021**; Nestle, 2018; Simpson, 2005	4
Belgium			1	TobaccoTactics, 2020	1
Chile			1	**Mialon, 2021**	1
Finland	1	Hiilamo, 2003			1
Germany	2	Bornhäuser *et al*., 2006; Schneider and Glantz, 2008;			2
Netherlands	1	Willemsen and Fooks, 2019			1
New Zealand			2	Hager, 2014; Espiner, 2021	2
Switzerland	2	Sasson, 2016; Anaenene, 2013	1	Chatterjee, 2013	2
United Kingdom			1	Campbell, 2012	1
United States	16	Balbach *et al*., 2000; Bialous *et al*., 2001; Drope and Chapman, 2001; Malone, 2002; Landman *et al*., 2002; Ibrahim *et al*., 2004; White and Bero, 2004; Carlini *et al*., 2006; McDaniel *et al*., 2006; Ibrahim and Glantz, 2006; Ibrahim and Glantz, 2007; Yang and Malone, 2008; Landman and Glantz, 2009; Thomson, 2009; Fallin and Glantz, 2015; McDaniel and Malone, 2020	9	Boseley, 2003 CCF, 2004; 2010; Pollan, 2006; Freudenberg, 2007; Heisel, 2011; Taubes and Kearns Couzens, 2012; Huehnergarth, 2016; Lorenzo, 2019	25
Regions that cannot be classified by income					
Europe	1	Adamini *et al*., 2011			1
Global	3	Smith and Malone, 2007; Brownell and Warner, 2009; Johnson, 2020	1	Daube, 2015	3
Total number^b^	41		28		69

^a^Islamic countries named in the article were: Algeria, Bangladesh, Egypt, Indonesia, Nigeria, Pakistan (LMICs) and Malaysia (UMIC) and Saudi Arabia (HIC), it is not clear which countries the intimidations occurred in. We've included the term under the LMIC banner as most countries mentioned were LMICs

^b^Number of articles that cover incidences of intimidation in different countries. Some articles in the literature cover more than one country and therefore appear more than once. Several articles include more than one instance of intimidation.

Articles (in bold) discuss intimidations in more than one country.

**Table 2:** Number of sources that include instances of intimidation, by intimidation type and industry sector

	Number of sources by sector												
Type of intimidation	Tobacco		UPF		SSB		Alcohol		Sector unclear		Total		
	PR	Grey	PR	Grey	PR	Grey	PR	Grey	PR	Grey	PR	Grey	All
Public discreditation total^a^	16	3	1	9	2	1	2	4	0	0	20	12	**32**
Traditional media^b^	9 (Drope and Chapman, 2001; Landman *et al*., 2002; Ibrahim *et al*., 2004; Bornhäuser *et al*., 2006; Smith and Malone, 2007; Schneider and Glantz, 2008; Landman and Glantz, 2009; Thomson, 2009; Patanavanich and Glantz, 2021)	0	1 (Baker *et al*., 2021)	3 (Pollan, 2006; Heisel, 2011; Taubes and Kearns Couzens, 2012)	2 (Vedwan, 2007; Johnson, 2020)	0	0	2 (Foundation for Alcohol Research and Education, 2017; RESYST, 2019)	0	0	12	5	**17**
Social media^c^	1 (Patanavanich and Glantz, 2021)	3 (Hager, 2014; Daube, 2015; Espiner, 2021)	0	5 (CCF, 2004, 2010; Hager, 2014; Espiner, 2021; Mialon, 2021)		**1 (** **Hager, 2014**)	0	2 (Hager, 2014; Espiner, 2021)	0	0	0	0	**12**
Other^d^	8 (Malone, 2002; Hiilamo, 2003; Knight and Chapman, 2004; White and Bero, 2004; Schneider and Glantz, 2008; Yang and Malone, 2008; Petticrew *et al*., 2015; Hoe *et al*., 2021)	0	0	1 (Peres, 2017)	0	0	2 (Avery *et al*., 2016; Matanje Mwagomba *et al*., 2018)	0	0	0	0	0	**11**
Legal threats/action	9 (Bialous *et al*., 2001; Hiilamo, 2003; Ibrahim *et al*., 2004; White and Bero, 2004; Ibrahim and Glantz, 2006, 2007; McDaniel *et al*., 2006; Landman and Glantz, 2009; Goel *et al*., 2021)	0	2 (Anaenene, 2013; Sasson, 2016)	1 (Mialon, 2021)	1 (Vedwan, 2007)	1 (Jacobs and Richtel, 2017)	0	0	0	0	12	2	**14**
Complaints to government bodies/international organizations/national and international authorities	7 (Bialous *et al*., 2001; White and Bero, 2004; McDaniel *et al*., 2006; Assunta *et al*., 2017; Egbe *et al*., 2019; Willemsen and Fooks, 2019; Patanavanich and Glantz, 2021)	0	1(Brownell and Warner, 2009)	0	0	1 (Jacobs and Richtel, 2017)	0	0	0	0	8	1	**9**
Complaints to the individual or organization	0	0	1 (Baker *et al*., 2021)	3 (Boseley, 2003; Freudenberg, 2007; Heisel, 2011)	0	1 (Lorenzo, 2019)	0	0	0	1 (Mialon, 2021)	1	5	**6**
Surveillance	5 (Malone, 2002; Knight and Chapman, 2004; White and Bero, 2004; Carlini *et al*., 2006; McDaniel *et al*., 2006)	1 (Simpson, 2005)	0	1 (Chatterjee, 2013)	0	3 (Huehnergarth, 2016; Jacobs and Richtel, 2017; Nestle, 2018)	0	0	0	1 (Mialon, 2021)	5	5	**10**
Freedom of information requests	6 (White and Bero, 2004; Carlini *et al*., 2006; Ibrahim and Glantz, 2006, 2007; Fallin and Glantz, 2015; Goel *et al*., 2021)	1 (Hager, 2014)	0	1 (Hager, 2014)	0	1 (Hager, 2014)	0	1 (Hager, 2014)	0	1 (Mialon, 2021)	6	2	**8**
Physical threats/violence	2 (Dharma N Mejia *et al*., 2008; Bhatta *et al*., 2020)	2 (Campbell and Meikle, 2012; McNeil, 2018)	0	0	0	2 (Jacobs and Richtel, 2017; Lorenzo, 2019)	0	0	0	0	2	4	**6**
Bribery	3 (Dharma N Bhatta *et al*., 2020; D. N. Bhatta *et al*., 2020; Goel *et al*., 2021)	0	0	0	0	0	0	0	0	0	3	0	**3**
Cyberattacks	0	0	0	0	0	2 (Jacobs and Richtel, 2017; Mialon, 2021)	0	0	0	0	0	2	**2**
Theft/burglary	0	1 (TobaccoTactics, 2020)	0	0	0	0	0	0	0	0	0	1	**1**
Other or unspecified threat/intimidation	8 (Balbach *et al*., 2000; Hiilamo, 2003; Adamini *et al*., 2011; Charoenca *et al*., 2012; Fallin and Glantz, 2015; McDaniel and Malone, 2020; Goel *et al*., 2021; Hoe *et al*., 2021 )	1 (Mialon, 2021)	0	1 (Mialon, 2021)	0	0	0	0	0	0	8	1	**9**

^a^Totals add up to more than the total number of articles in the review as some of the grey literature, the articles (in bold), discuss more than one industry. For the public discreditation, total score we counted the total unique sources overall even though some sources listed more than one public discreditation type (e.g. social media and other or traditional media and social media). Where a source included the same type of intimidation on several different targets, this is only counted once.

^b^includes discreditation through traditional media such as print newspapers and online statements on websites.

^c^Social media websites such as Facebook.

^d^Other methods of public discreditation including public consultation responses, t-shirts with discreditation campaigns or unspecified methods of public discreditation.

### Types of intimidation

We identified 10 main forms of intimidation and several instances of intimidation that did not fit into the 10 categories and were categorized as ‘other’ (Table 2). We built on the previous framework by Matthes *et al*. (2022) and Matthes *et al*. (2023), we added one new category (bribery) and did not use two pre-existing ones (non-anonymous intimidating messages and anonymous intimidating messages) (Supplementary File S1).

#### Public discreditation

The most common form of intimidation found in half of the included sources and across all sectors was public discreditation (*n* = 32; 50%) (see Supplementary File S2 for a full list of interesting quotes). Academics, as well as advocates and their work, were publicly criticized in traditional media (newspapers, magazines, advertisements, op-eds, editorials, public statements, responses to journal articles, organization websites, press releases), on social media and in other public fora such as evidentiary meetings, public consultation responses, tv interviews and even on t-shirts in one documented incident.

Much of the discreditation focused on undermining individuals and organizations. Researchers were publicly labelled as ‘extremists’ (Landman *et al*., 2002; Malone, 2002; Knight and Chapman, 2004; Yang and Malone, 2008; Avery *et al*., 2016; Johnson, 2020), ‘fascists’ (Smith and Malone, 2007), ‘nazis’ (Schneider and Glantz, 2008), ‘zealots’ (Daube, 2015), ‘demons of overzealous and moral righteousness’ (Knight and Chapman, 2004) and ‘prohibitionists’ (Landman and Glantz, 2009; Daube, 2015). Similarly, those producing research unfavourable to the alcohol industry were labelled ‘nannyists’ (Avery *et al*., 2016), those doing the same in the UPF sector were called ‘too radical’ (Mialon, 2021), ‘food fascists’ (CCF, 2010), ‘gastronomical gestapo’ (Thomson, 2009) and ‘food police’ (Heisel, 2011), while breastfeeding advocates were described as the ‘breastapo’ (Hager, 2014) and were portrayed as ‘limiting mothers’ freedom of choice’ (Baker *et al*., 2021). Negative religious connotations were also used across the different industry sectors, with academics referred to as the ‘anti-food jihad’ (CCF, 2004), ‘health jihadists’ (Hager, 2014) and ‘religious fundamentalists’ (Petticrew *et al*., 2015; RESYST, 2019).

In addition, across all sectors, researchers were criticized as lacking the relevant skills (Drope and Chapman, 2001; Landman and Glantz, 2009; RESYST, 2019), being ‘bogus’ (Landman and Glantz, 2009), ‘untrustworthy’ (Vedwan, 2007), ‘mad’ (Hager, 2014), having conflicts of interest (Ibrahim *et al*., 2004), being money hungry (White and Bero, 2004; Landman and Glantz, 2009; Hoe *et al*., 2021), ‘publicly funded troughers’ (Hager, 2014) or simply for being ‘peculiar’ (Bornhäuser *et al*., 2006) or for not having the right physique to criticize the food industry (Pollan, 2006).

Other attempts at discreditation in each of the sectors focused on undermining scientific research by paying other scientists and physicians to present conflicting evidence (Hiilamo, 2003; Vedwan, 2007; Peres, 2017; Matanje Mwagomba *et al*., 2018; Mialon, 2021; Patanavanich and Glantz, 2021), stating that no evidence existed when it did (Taubes and Kearns Couzens, 2012; Foundation for Alcohol Research and Education, 2017), launching online campaigns to oppose experts in the field (Patanavanich and Glantz, 2021) and questioning the motives of funders (RESYST, 2019), researchers (Hager, 2014; Daube, 2015; Hoe *et al*., 2021) and advocates (Hager, 2014). A key argument was that government/taxpayers’ money was being used to unlawfully lobby the government on public health (Drope and Chapman, 2001; White and Bero, 2004; Landman and Glantz, 2009; Hager, 2014).

Most articles detailing public discreditation identified the perpetrators as industry-linked third parties, across the tobacco, UPF and alcohol sectors (*n* = 23, 71.9%). Third parties acting on behalf of industry included front groups (tobacco, alcohol, UPF) (White and Bero, 2004; Pollan, 2006; Smith and Malone, 2007; Schneider and Glantz, 2008; Landman and Glantz, 2009; Heisel, 2011; Avery *et al*., 2016; Matanje Mwagomba *et al*., 2018), industry-funded politicians (tobacco) (Ibrahim *et al*., 2004), lobbyists (tobacco, UPF) (Ibrahim *et al*., 2004; Baker *et al*., 2021), industry representatives (tobacco, alcohol) (Bornhäuser *et al*., 2006; Matanje Mwagomba *et al*., 2018), paid scientists and consultants (tobacco, alcohol, UPF) (Hiilamo, 2003; Hager, 2014; Peres, 2017; Espiner, 2021) or more than one of these (Daube, 2015). In nine articles (28.1%), corporations acting in their own name across each sector (either alone or alongside industry-linked third parties) undertook public discreditation activities (Vedwan, 2007; Yang and Malone, 2008; Landman and Glantz, 2009; Petticrew *et al*., 2015; Foundation for Alcohol Research and Education, 2017; RESYST, 2019; Johnson, 2020; Hoe *et al*., 2021; Mialon, 2021), while four articles (12.5%) referred to just the ‘industry’ as the perpetrators (Drope and Chapman, 2001; Landman *et al*., 2002; Malone, 2002; Knight and Chapman, 2004).

#### Legal threats and action

Fourteen articles (21.9%) discussed instances where industry and its third parties threatened, or brought legal action against researchers and advocates in the tobacco and UPF, including SSB sectors. There were no examples of legal threats in the scarcer alcohol sector literature.

In the 1990s in the USA, tobacco companies and their third parties took legal action to prevent or delay tobacco control legislation and initiatives such as mass media campaigns. For example, tobacco companies claimed that advocates were breaking the terms of the Master Settlement Agreement by denigrating the tobacco industry in mass media campaigns which then tied advocates up in legal threats and action for years despite eventually winning the cases and carrying on with their work (Ibrahim *et al*., 2004; White and Bero, 2004; Ibrahim and Glantz, 2006, 2007; McDaniel *et al*., 2006; Landman and Glantz, 2009). Similarly, in India, it was reported that the tobacco industry frequently used legal threats to delay tobacco control legislation and intimidate advocates and researchers (Goel *et al*., 2021). In Finland, the tobacco industry successfully managed to temporarily change the rules so that lawyers were personally responsible for settlement costs instead of the plaintiffs who were suing the tobacco industry. They did so to encourage lawyers to drop or refuse cases (Hiilamo, 2003).

The sources also included examples of legal action against advocates and researchers producing work unfavourable to the UPF industry (Vedwan, 2007; Anaenene, 2013; Sasson, 2016; Jacobs and Richtel, 2017; Mialon, 2021). In one instance, a Swiss advocacy group was successfully sued for libel after a food corporation objected to the translation of the title of a report written by the group (Anaenene, 2013; Sasson, 2016). In Colombia, a consumer advocacy group was legally prohibited from publicly discussing the detrimental health impacts of sugar and was subsequently sued for allegedly breaking this rule (Jacobs and Richtel, 2017). Similarly, researchers focusing on SSBs in Brazil were also threatened with legal action after they produced a large inflatable can of a well-known SSB brand with the word Diabetes on the side (Mialon, 2021). In India, the SSB industry took an advocacy group to court for a report stating that the beverages contained pesticides but later dropped the case as it ‘won’t improve our brand image’ (Vedwan, 2007). Others working on food-related research were threatened with legal action unless they withdrew their research press release ‘at the eleventh hour’ (Mialon, 2021).

#### Complaints to authorities

Nine sources in the dataset (14.1%) included examples of industry complaining to authorities about the work of researchers and advocates. Eight discussed complaints made to government agencies about tobacco advocacy and research (Bialous *et al*., 2001; White and Bero, 2004; McDaniel *et al*., 2006; Assunta *et al*., 2017; Jacobs and Richtel, 2017; Egbe *et al*., 2019; Willemsen and Fooks, 2019; Patanavanich and Glantz, 2021). The remaining article concerned the UPF sector and reported the threat that funding to the WHO would be cut unless researchers changed their recommendations (Brownell and Warner, 2009). No examples of complaints to authorities were apparent for the alcohol sector in our dataset.

The complaints included accusations of illegal lobbying for health policies in the USA (after the industry secured policy change which made lobbying by state-funded organizations illegal) (Bialous *et al*., 2001; White and Bero, 2004; McDaniel *et al*., 2006; Assunta *et al*., 2017), too much access and power given to advocacy groups (Nigeria) (Egbe *et al*., 2019), inappropriate handling of finances and tax avoidance (US) (Bialous *et al*., 2001; McDaniel *et al*., 2006), presenting untrue or misleading science (tobacco and UPF) (Brownell and Warner, 2009; Jacobs and Richtel, 2017) or unfair practices limiting industry from ‘legitimate conversations’ with policy-makers (Netherlands) (Willemsen and Fooks, 2019).

These complaints came from the industries themselves (Assunta *et al*., 2017; Jacobs and Richtel, 2017; Willemsen and Fooks, 2019; Patanavanich and Glantz, 2021) and industry-funded third parties (Bialous *et al*., 2001; White and Bero, 2004; Brownell and Warner, 2009; Egbe *et al*., 2019), as well as industry-linked politicians (McDaniel *et al*., 2006).

#### Complaints to the individual or organization

A further six sources (9.4%) included examples where advocates and researchers working on issues affecting UPF, and in one case tobacco, received a complaint. These complaints took several forms, including a detailed critique of research and a request for the original data for ‘proper’ analyses to be conducted (Freudenberg, 2007), repeated requests for the tax reporting of an advocate (Heisel, 2011) and emails trying to get a researcher to stop their work or change their findings (Boseley, 2003; Mialon, 2021). These three instances were all undertaken by UPF industry-funded third parties. In another instance, an industry body complained in person and threatened to block the publication of a report on the harmful impacts of sugar unless the authors diluted its public health recommendations (Boseley, 2003). The fifth reported how an advocate and researcher campaigning for an increase in soda taxes was asked by an SSB company not to attend its annual shareholder meeting despite originally inviting them (Lorenzo, 2019).

Finally, UNICEF’s Director General received complaints from a US-based lobby group working for the baby formula industry. The complaints argued that UNICEF’s Philippines country office promoting breastfeeding ‘misrepresents the available scientific evidence regarding the alleged risks of not-breastfeeding’. Complaints were also made to UNICEF’s regional offices (Baker *et al*., 2021).

#### Surveillance

Ten sources (15.6%) described instances where researchers or advocates had been watched. Four detailed the extensive surveillance that tobacco control advocates experienced in the 1980s, 1990s and early 2000s (Malone, 2002; White and Bero, 2004; Carlini *et al*., 2006; McDaniel *et al*., 2006). ‘Aggressive intelligence gathering’ was used, including sending employees or third parties to gather materials at tobacco control meetings ‘under false pretences’ (Malone, 2002; White and Bero, 2004). The ‘common ground database’ of individuals and organizations considered to be anti-smoking was produced and was ‘searchable by tobacco control issues, groups, people…’ (Malone, 2002; Carlini *et al*., 2006; McDaniel *et al*., 2006). While most of the evidence refers to the USA, similar instances occurred in Asia and Australia, including third parties going through the bins of ‘anti-smoking’ organizations (Knight and Chapman, 2004; Simpson, 2005).

In the UPF sector, in Switzerland in 2003, a corporation hired a firm to spy on an advocacy group that was writing a book on its corporate practices (Chatterjee, 2013; Mialon, 2021). These spies attended meetings, even those in private homes. One source suggested that undercover attendance and covert recordings of public health meetings were commonplace (Mialon, 2021). In Colombia, a member of a public health advocacy organization was followed, and computers and phones were allegedly bugged (Jacobs and Richtel, 2017). This type of phone bugging also occurred to advocates working on SSB tax increases in Mexico (see *Cyberattack* section) (Jacobs and Richtel, 2017). In Australia, a talk by a public health researcher was watched by an SSB company representative who subsequently recommended to the corporation that the researcher be put under increased surveillance (Huehnergarth, 2016; Nestle, 2018). In another instance, those working on content unfavourable to the industry (sector and country not disclosed) received anonymous intimidating phone calls where the callers said they were following their children home from school (Mialon, 2021). While we know in the USA and Swiss cases that corporations and their third parties were behind the surveillance, it is not known who was responsible for the instances in Latin America.

#### Freedom of information requests

Eight sources (12.5%) included instances where FOI requests had been used to intimidate advocates and researchers across the sectors. Six of these described the use of FOI against those working in tobacco control in the USA (White and Bero, 2004; Carlini *et al*., 2006; Ibrahim and Glantz, 2006, 2007; Fallin and Glantz, 2015) and India (Goel *et al*., 2021). In the USA, tobacco companies, lawyers, front groups, industry lobbyists and consultants all filed requests for documents to thwart tobacco control progress (White and Bero, 2004; Carlini *et al*., 2006; Ibrahim and Glantz, 2006, 2007), delaying advocates’ work and ‘providing a roadmap that the industry used to plan its attacks’ (Fallin and Glantz, 2015). This has also happened to academics working in the UPF sectors (Mialon, 2021). The eighth source described how an industry consultant working for corporations across the sectors consistently used FOI requests to gather intelligence on those advocating for tobacco, alcohol and infant formula regulations in New Zealand before using the information in defamatory blog posts (Hager, 2014).

#### Actual or threats of physical violence

Six sources (9.4%) described threats of, or actual physical violence towards those advocating for increased regulatory measures for tobacco and SSBs. These instances occurred in Latin America (Argentina, Colombia and Mexico) (Mejia *et al*., 2008; Jacobs and Richtel, 2017; Lorenzo, 2019), Asia (Nepal and Indonesia) (McNeil, 2018; Bhatta *et al*., 2020), Africa (Nigeria) (McNeil, 2018) and Europe (UK) (Campbell and Meikle, 2012).

In the most extreme case, in Nigeria in 2012, following media appearances criticizing the tobacco industry, a leading tobacco control advocate and his children were threatened at gunpoint and two men were shot and killed (McNeil, 2018). Despite this event occurring following these media appearances, it is not known who organized the house invasion. In Indonesia, leading tobacco control advocates were pictured on wanted posters and one was threatened at his home in front of his children and told to leave the country (McNeil, 2018). In Nepal, those advocating for graphic health warnings on tobacco packs received death threats (Bhatta *et al*., 2020), as did those advocating for SSB tax increases in Colombia and Mexico (Jacobs and Richtel, 2017; Lorenzo, 2019). In Colombia, an SSB tax advocate was followed on more than one occasion and told to ‘shut her mouth’ (Jacobs and Richtel, 2017). In Argentina, newspaper articles included personal threats to tobacco control advocates (Mejia *et al*., 2008). In the UK, in response to standardized packaging proposals and research which exposed tobacco industry conduct, researchers and advocates were threatened with violence via intimidatory phone calls and blog posts inciting others to commit violence against them (Campbell and Meikle, 2012). It was not possible to determine whether any of these instances were linked to a corporation.

#### Bribery

Three sources (4.7%) referred to bribery, two in Nepal and one in India, all involving tobacco. Tobacco control advocates were offered ‘money’ (Bhatta *et al*., 2020), ‘other support (whatever they liked)’ (Bhatta *et al*., 2020) and ‘undue favours’ (Goel *et al*., 2021) to ‘remain inactive in the policy-making process’ (Bhatta *et al*., 2020) or to ‘interfere with the implementation of tobacco control laws’ (Goel *et al*., 2021) and to leave tobacco companies and their business alone (Goel *et al*., 2021). Bribes were offered to tobacco control advocates in Nepal at two different time points, before a tobacco control bill was passed in 2010 and then again when the industry challenged the adopted tobacco control regulations in the Supreme Court. Despite these bribes being offered for these specific purposes, it was not clear who was responsible.

#### Cyberattacks

Linked to surveillance and threats of violence, two sources (3.1%) detailed alleged cyberattacks in Latin America (Colombia and Mexico) (Jacobs and Richtel, 2017; Mialon, 2021). Spyware was reportedly found on the mobile phones of advocates working in the SSB sector and the office router and several computers of employees at a public health advocacy agency and staff reported that its IT networks were infiltrated and data changed (Jacobs and Richtel, 2017; Mialon, 2021). Nobody was found culpable.

#### Burglaries

One source (1.6%) discussed an incident where tobacco control NGOs in Brussels were burgled when the revision of the European Union’s Tobacco Products Directive was being debated (TobaccoTactics, 2020). Although no one has ever been apprehended, laptops containing confidential digital files, as well as printed documents relevant to the Directive were taken, suggesting a targeted sweep.

#### Other

Nine further sources (14.1%) described forms of intimidation that could not be categorized in the amended or pre-existing framework of intimidation or did not include enough information for the intimidation to be definitively categorized. Eight sources referred to the tobacco sector, and one source referred to the UPF sector, including one which referred to both. Unspecified intimidations included mentions of ‘threats’ and ‘aggressive attacks’ and ‘pressure’ levied against tobacco control advocates (Balbach *et al*., 2000; Charoenca *et al*., 2012; Goel *et al*., 2021; Hoe *et al*., 2021) or cited advocates or researchers as the number one target of the industry but without specifying how they were targeted (Adamini *et al*., 2011).

Other forms of intimidation included attempts to co-opt tobacco control researchers (Hiilamo, 2003; Mialon, 2021), thwarting research progress by holding a research paper for 9 months before finally rejecting it without peer review (the submitting author later found out there was a link between the editor of the journal and the industry) (Mialon, 2021), flooding a tobacco control help phone line with spurious calls (Fallin and Glantz, 2015) and mobilizing hundreds of people to attend a tobacco control meeting to object to a proposed ban on tobacco sales in the area (McDaniel and Malone, 2020). One researcher reported trying to stay out of sight in a tobacco industry stronghold town in Brazil while discussing the WHO Framework Convention on Tobacco Control (WHO FCTC) with local tobacco farmers for fear of how the industry might react (Mialon, 2021). Tobacco control advocates in Thailand were pressured not to release information about tobacco ingredients to the public (Charoenca *et al*., 2012).

### Impacts of intimidation

Over a third of sources (*N* = 24; 37.5%) did not discuss the impacts of intimidation (Landman *et al*., 2002; Hiilamo, 2003; CCF, 2004, 2010; Simpson, 2005; McDaniel *et al*., 2006; Smith and Malone, 2007; Vedwan, 2007; Schneider and Glantz, 2008; Yang and Malone, 2008; Brownell and Warner, 2009; Thomson, 2009; Adamini *et al*., 2011; Anaenene, 2013; Chatterjee, 2013; Hager, 2014; Petticrew *et al*., 2015; Avery *et al*., 2016; Huehnergarth, 2016; Matanje Mwagomba *et al*., 2018; Lorenzo, 2019; McDaniel and Malone, 2020; TobaccoTactics, 2020; Hoe *et al*., 2021).

For those sources that did, the impacts were multifaceted, affecting advocacy and research activities and individuals across every sector in this review.

The dominant theme in the literature was that intimidation had a chilling effect on the work of advocates and researchers and, therefore, the passage and implementation of policy. **Legal threats and action, complaints, public discreditation and FOIs** reduced resources available for the work while those targeted responded or raised a defence (Bialous *et al*., 2001; White and Bero, 2004; Carlini *et al*., 2006; Ibrahim and Glantz, 2006, 2007; Freudenberg, 2007; Landman and Glantz, 2009; Heisel, 2011; Egbe *et al*., 2019; RESYST, 2019; Johnson, 2020; Mialon, 2021). **Cyberattacks** meant that individuals were physically unable to do their work because their ‘*laptops would stop following orders and the mouse would just do what it wanted*’ (Jacobs and Richtel, 2017). **Legal action** meant that some advocates were prevented from pursuing hard-hitting policies such as smoke-free environments (Ibrahim *et al*., 2004) whilst others were prevented from publishing accurate data on sugar consumption (Boseley, 2003; Taubes and Kearns Couzens, 2012; Jacobs and Richtel, 2017) and tobacco ingredients (Charoenca *et al*., 2012). **Bribes** were offered to tobacco control NGOs to become passive in a supreme court case in Nepal, and, although it is not known whether the bribes were accepted, one NGO did become silent. Similarly, **personal threats** to tobacco control advocates in Argentina, in conjunction with other CPA, reportedly led an Argentinean province to recommend that the country avoid ratifying the WHO FCTC (Mejia *et al*., 2008). To date, the country still has not ratified the Treaty. The individual contribution of the threats to this outcome remains unclear.

Other times advocates were intimidated enough by **complaints and interference** (flooding ASSIST call lines) to start pursuing individual-level policies that were less threatening to the industry than population-level regulatory measures (Bialous *et al*., 2001; Fallin and Glantz, 2015), and one academic reported that co-authors of academic papers removed their names out of fear of being sued (Mialon, 2021). Another source reported a negative impact on the reputation of a health advocacy group following industry complaints to the government (Assunta *et al*., 2017).

The sources also revealed that intimidation was facilitated by industry success in pushing for amended lobbying rules in the USA and Nigeria, which allowed the industry to continually complain about ‘unlawful lobbying’ by health advocates and stopped some advocates from their usual activities (Bialous *et al*., 2001; White and Bero, 2004; Egbe *et al*., 2019). Similarly, some instances of **public discreditation** successfully ‘marginalized’ health advocates and influenced decision-makers’ perceptions (Bornhäuser *et al*., 2006; Taubes and Kearns Couzens, 2012).

In many cases, regulations were passed, albeit significantly delayed, for example, tobacco control legislation in the USA (Bialous *et al*., 2001; Drope and Chapman, 2001; Bornhäuser *et al*., 2006), Asia (Knight and Chapman, 2004), including Nepal (Bhatta *et al*., 2020) and India (Goel *et al*., 2021), and UPF regulations in the Philippines (Baker *et al*., 2021).

As well as having impacts on the work, intimidatory behaviour impacted individuals’ wellbeing. **Public discreditation** was reported as ‘untrue, unfair, offensive, insulting and defamatory’ (Espiner, 2021) ‘wearying’, ‘unpleasant’, ‘intimidating’ and ‘distressing’ (Daube, 2015), whilst threatening phone calls and threats of violence led the recipients to feel ‘worried’ and ‘threatened’ (Campbell and Meikle, 2012). In Latin America, an advocate reported feeling ‘extremely frustrated’ that the industry could say what it wanted but that advocates could not report the truth about sugar (Mialon, 2021). Financial impacts included an advocacy group being successfully sued by a corporation for libel (Anaenene, 2013; Sasson, 2016), another spending $20 000 USD of their own money (Ibrahim and Glantz, 2006), and an advocate facing financial ruin if an industry lawsuit against them were successful (Jacobs and Richtel, 2017). In the most extreme case in Nigeria, the brother-in-law and the house guard of a tobacco control advocate were shot and killed during a house raid (Johnson, 2020).

### Responses to intimidation

We identified four types of responses to intimidation in line with Matthes *et al*.’s (2023) framework (Matthes *et al*., 2023). We made one change to the name of one of the categories. ‘No action’ was renamed to ‘Carry on as planned’ as it was felt that ‘no action’ inadvertently suggested a passive response to intimidation, whereas ‘carrying on’ was a more accurate representation of the active role being taken by those who continue with their work despite being intimidated (Supplementary File S3). It is possible to react in multiple ways.

Withdrawal (i.e. stopping work in an area because of intimidation): Following legal threats and action some advocates were reported to be wary of engaging in certain types of work (Bialous *et al*., 2001), others wanted their names removed from publications (Mialon, 2021), and some resigned (Ibrahim *et al*., 2004). Following public discreditation on social media one individual reported that they would no longer work on a particular tobacco control topic because they didn’t have ‘the stomach for the gross Twitter trolls that descended or the internet vermin that took over the comments’ (Daube, 2015).

Carry on as planned: In several instances, researchers and advocates defiantly carried on with their work despite intimidation (Knight and Chapman, 2004; Freudenberg, 2007; Daube, 2015; Lorenzo, 2019; Mialon, 2021; Patanavanich and Glantz, 2021). For example, a leading advocate refused to buy a new phone following a cyberattack (Jacobs and Richtel, 2017), another refused to leave the country following death threats (McNeil, 2018), and another reported that they assumed that the industry always knew what they planned to say so they did not worry about self-censoring (Mialon, 2021). In India, an academic reported turning down industry offers of funding (Goel *et al*., 2021). Whilst some sources explicitly referred to carrying on with their work, in other cases, this was not explicitly stated but the defensive and offensive actions they took implied that they also carried on with their work, and so the majority of instances reported in the next two sections also carried on with their work.

Defensive action: Several sources reported individuals taking at least some measures to amend their work or behaviour to protect themselves from further intimidation, such as increased security (Campbell and Meikle, 2012; Jacobs and Richtel, 2017; McNeil, 2018), ‘abandoning’ their home (McNeil, 2018), stopping driving alone (Jacobs and Richtel, 2017), changing the focus of the work to less effective interventions (Bialous *et al*., 2001; Ibrahim *et al*., 2004; Fallin and Glantz, 2015), e.g. smoking cessation programmes instead of indoor smoke-free policies (Ibrahim *et al*., 2004), self-censorship (Bialous *et al*., 2001; Boseley, 2003; White and Bero, 2004), using encrypted apps for communications instead of email and phone (Jacobs and Richtel, 2017) and production of more facts to support statements (Peres, 2017). Advocates leading a boycott of Nestlé in the 1980s had to issue statements defending the legitimacy of their boycott after the company claimed it had changed for the better (Johnson, 2020). In the USA, those working in tobacco control made exhaustive attempts to keep the industry out of their meetings, for example, suggesting that attendees sit in a circle and scan the room for faces they did not recognize (Malone, 2002).

Offensive action: Many sources reported examples of offensive action. This included advocates and researchers correcting misinformation (Bialous *et al*., 2001; Ibrahim *et al*., 2004; Heisel, 2011; Baker *et al*., 2021; Hoe *et al*., 2021; Patanavanich and Glantz, 2021), exposing industry conduct in the media (Balbach *et al*., 2000; Bialous *et al*., 2001; Ibrahim *et al*., 2004; Pollan, 2006; Landman and Glantz, 2009; Heisel, 2011; Jacobs and Richtel, 2017; Baker *et al*., 2021), openly criticizing the government for allowing the industry to manipulate their opinions (Bornhäuser *et al*., 2006), remaining critical of a UPF company whilst being sued by them (Sasson, 2016), calling the police (Campbell and Meikle, 2012; Jacobs and Richtel, 2017; McNeil, 2018; TobaccoTactics, 2020) and taking legal action (Balbach *et al*., 2000; Ibrahim and Glantz, 2006, 2007; Chatterjee, 2013; Willemsen and Fooks, 2019; Espiner, 2021). In some cases, other advocacy organizations came to the aid of those being publicly denigrated and vouched for their work (Ibrahim *et al*., 2004; Landman and Glantz, 2009; Baker *et al*., 2021). One researcher/advocate reported attending a lobbying course intended for corporate lobbyists in order to understand their behaviour and ‘their strategies’ (Mialon, 2021). One expert published a list of locations and times of her talks after learning that she was under surveillance (Huehnergarth, 2016; Nestle, 2018).

### Outcomes of advocates or researchers’ responses

When advocates and researchers respond to intimidation, very little information on the outcomes is provided. Even where information on policy outcomes and legal rulings is available, it is not possible to quantify the role of researchers’ and advocates’ activities.

In some cases, after taking offensive actions such as correcting misinformation and providing research evidence, legal cases against researchers and their institutions were dropped or dismissed (Bialous *et al*., 2001; Freudenberg, 2007; Landman and Glantz, 2009). A censorship order was overturned (Jacobs and Richtel, 2017; Lorenzo, 2019; Mialon, 2021), tobacco control programmes were reinstated (Ibrahim and Glantz, 2006), regulations passed in the USA (Balbach *et al*., 2000), the Philippines (Baker *et al*., 2021; Hoe *et al*., 2021) and Ukraine (Hoe *et al*., 2021) and attempts to interrupt funding streams for tobacco control activities were defeated (Balbach *et al*., 2000; Ibrahim and Glantz, 2007). A ban on newer nicotine and tobacco products in Thailand remained in place despite industry attempts to revoke it (Patanavanich and Glantz, 2021).

In New Zealand, a defamation case brought by researchers and advocates working in tobacco, alcohol and food was successful against an industry-paid consultant who confessed that his behaviour had been a deliberate and untrue industry-funded campaign to suit the needs of those who were paying him (Espiner, 2021). A Swiss advocacy group also successfully sued a corporation for spying on them (Chatterjee, 2013). Reputational damage to the same corporation was achieved by advocates during the 2-year libel case despite the corporation winning the case (Sasson, 2016). In the Netherlands, tobacco control advocates lost their legal challenge against the government for violation of WHO FCTC Article 5.3 but the ultimate outcome was positive because the government subsequently limited interactions with the tobacco industry (Willemsen and Fooks, 2019).

In all instances where the police were called for threats of violence, actual violence or burglary, no arrests were reported (Campbell and Meikle, 2012; Jacobs and Richtel, 2017; McNeil, 2018; Lorenzo, 2019). Despite the attempts to keep the industry out of tobacco control meetings, industry spies still infiltrated them and made recordings (Malone, 2002). A food advocacy organization’s attempt to hold a boycott against a corporation failed, ‘leaving the [advocacy] organization demoralized and heavily in debt’ (Johnson, 2020).

## Discussion

While existing literature has highlighted intimidatory tactics as part of UCI CPA (Ulucanlar *et al*., 2023), this study is the first to explore intimidation across the tobacco, alcohol and UPF sectors in more detail. We found ample evidence of similar intimidation experienced by advocates and researchers across the sectors. There was significantly less information available on the behaviour of the alcohol industry but it is unclear whether this is a true representation of the alcohol industry’s behaviour or an absence of evidence in the literature we reviewed. Further qualitative research with advocates and researchers working in the alcohol sector is warranted to explore this further.

Intimidation had a chilling effect on the work of advocates and researchers regardless of how they responded. However, although it is not possible to determine causality, many of those who took action to counter the intimidatory tactics or who carried on a usual with their work, albeit delayed, were associated with policy success stories.

The perpetrators of the intimidation were mostly either the corporations themselves or third parties with industry links. However, in the more extreme forms of intimidation such as threats of violence, and actual incidences of violence, cyberattacks and surveillance, the perpetrators were unknown. This may be because the perpetrators are genuinely unknown or that fear of retribution has prevented their disclosure.

The most reported form of intimidation was public discreditation with advocates and researchers often portrayed as extremists, under-qualified, or a waste of taxpayer money. Spreading such perceptions may limit researchers’ and advocates’ ability to achieve evidence-based policy impact, an explicit intention of PMI (McDaniel *et al*., 2006; Ulucanlar *et al*., 2016). Public discreditation was described as wearying and unpleasant, yet most sources did not mention how experiences of public discreditation made individuals feel or what other impacts it had. For those who took offensiv action, such as outing the intimidation, legal action or correcting misinformation, it was implied that these individuals also carried on with their work (Bornhäuser *et al*., 2006; Landman and Glantz, 2009; Johnson, 2020; Baker *et al*., 2021; Hoe *et al*., 2021; Patanavanich and Glantz, 2021), however only a handful of sources explicitly confirmed this (Malone, 2002; Knight and Chapman, 2004; Patanavanich and Glantz, 2021).

Legal threats and actions, the second most identified form of intimidation here, were successful in delaying the passage and implementation of tobacco control and SSB mass media campaigns and the publication of academic work, even when the legal action was unsuccessful (White and Bero, 2004; Ibrahim and Glantz, 2006; McDaniel *et al*., 2006; Schneider and Glantz, 2008; Landman and Glantz, 2009; Jacobs and Richtel, 2017; Goel *et al*., 2021; Mialon, 2021). Legal challenges are costly, and those without organizational support may face personal financial ruin in such circumstances. However, legal challenges did appear to come at a reputational cost for some corporations in the long term (e.g. Nestlé) (Anaenene, 2013; Sasson, 2016). Other corporations who recognized this reputational risk dropped their legal challenge against advocates (Vedwan, 2007). FOIs and complaints were also identified as extremely disruptive by advocates and researchers (White and Bero, 2004; Landman and Glantz, 2009; Johnson, 2020; Mialon, 2021).

More serious forms of intimidation, such as cyberattacks, surveillance, burglaries and threats of violence, and actual violence were less frequently reported in the literature than other types of intimidation. Other than burglaries which occurred in Belgium, these all occurred in LMICs—in Latin America (cyberattacks, surveillance, perceived threats of violence), Asia (threats of violence) and Africa (actual violence). None were directly attributed to a corporation or industry, but all occurred in direct response to work that threatened UCIs. These forms of intimidation were predominantly discussed in what we defined as the grey literature (e.g. blogs, news stories, books, press interviews and webinars) and were often more detailed and candid than the instances described in the peer-reviewed literature. This is unsurprising as academics who have been intimidated are primarily concerned with publishing papers on the health impacts of consumable products, exploring policy influence and evaluating the impacts of regulations and do not have the additional resources necessary to write peer-reviewed papers on their experiences of intimidation. It may also be the case that journal editors are less likely to publish papers that focus solely on intimidation. Given this finding, a deeper and more systematic exploration of the grey literature may be warranted.

The findings of this review support a previously developed framework of tobacco industry intimidation based on survey and interview data (Matthes *et al*., 2022, 2023), with some small differences with bribery classified as its own category of intimidation (Tobacco Tactics, 2020). The previous framework included intimidating messages (both anonymous and non-anonymous) as intimidation types. We did not use these categories here as anything that could be conceivably categorized as such was categorized as a complaint, threat of violence or surveillance.

There was little information on the impacts of the intimidation, but findings suggested intimidation had a chilling effect on the policy process, including the implementation of public health interventions (e.g. anti-tobacco and anti-SSB mass media campaigns). Advocates and researchers felt frustrated and distressed at being temporarily silenced, their work delayed, or thwarted. Some were financially affected (Daube, 2015). Nevertheless, the dominant narrative was of perseverance, with only two sources referring to staff leaving their job and another individual potentially withdrawing from some of their usual advocacy activities (Daube, 2015). This contrasts with recent interview-based work where interviewees reported being driven out of tobacco control through intimidation (Matthes *et al*., 2022). Therefore, the lack of withdrawal found in this scoping review should not be interpreted as true evidence of absence. Rather, it is likely that such occurrences are not being captured in the literature because the individuals have left the field and are not reporting their experiences, or perhaps successful counter strategies are more likely to be reported. This highlights the need for qualitative research about intimidation with such individuals.

Of the few sources that did document responses to intimidation, most reported *offensive* action including exposing the intimidation, correcting false information and taking legal action. Many of these responses were associated with successful outcomes, e.g. passing of regulations, legal cases being dropped, campaigns reinstated, but causality cannot be determined. Nevertheless, these examples could embolden others experiencing intimidation (Matthes *et al*., 2023).

Those taking *defensive* action reported being more careful with what they said, what they published and actions they took outside work (e.g. increasing security). Some reported changing the focus of their work away from evidence-based population-level interventions (e.g. tax increases) to weaker interventions such as individual-level interventions (e.g. stop smoking services). One tobacco control expert recommended that health organizations and future leaders provide strong support to the next generations of campaigners so that they are prepared for the potential backlash they may face (Daube, 2015).

Given the similarity of intimidation experienced across the sectors, collaboration on how individuals and organizations might respond to such instances is warranted. Spreading awareness amongst colleagues is likely to be a helpful first step in this process. Support by communities in HICs, for colleagues in LMICs, may also be warranted due to resource limitations.

## Limitations

The overrepresentation in this study of the tobacco industry is likely due to the availability of internal industry documents made public in the late 1990s which facilitated research into tobacco industry conduct in the early 2000s, largely in the USA. This may also help explain why most of the intimidation included in this article occurred in HICs compared to LMICs, although LMICs feature more prominently over time and the most serious forms of intimidation, such as surveillance, cyberattacks and threats of violence.

This review focused primarily on the peer-reviewed literature, supplemented with a conservative amount of grey literature. Greater resources to explore more of the grey literature would have undoubtedly found more instances of intimidation. However, our purpose was not to examine the scale but to identify the types of intimidation across the sectors of interest. Yet, given that more extreme forms of intimidation were largely found in the grey literature there is a rationale for exploring this literature further.

Due to resource constraints, we did not explore intimidation in other sectors (Bennett *et al*., 2022) such as pharmaceuticals, human rights, environment and climate change, where more extreme forms of intimidation have been documented in relation to large corporations (Knight and Greenberg, 2011; Bell *et al*., 2019; Hudson, 2020; Busch and Judick, 2021).

## Conclusion

Advocates and researchers experienced intimidation which delayed, derailed and prevented regulation of UCIs across all country income groups and across all the examined sectors. Each type of intimidation led to adverse impacts on the work yet only little information is available on how it impacted the individuals intimidated. It is important for the global public health community to be aware of the ways in which they may be intimidated for their work so that they can develop resilience through support networks and tried and tested counter strategies. Information on how advocates and researchers responded to intimidation, while sparse, found that offensive action was associated with positive—although often delayed—policy outcomes. More work is needed to explore the relationship between the effectiveness of responses as ‘such an understanding may go some way to diffusing the effectiveness of the attacks’ (Knight and Chapman, 2004).

## Supplementary Material

daae153_suppl_Supplementary_Files_1

daae153_suppl_Supplementary_Files_2

daae153_suppl_Supplementary_Files_3

## Data Availability

All papers included in the review are publicly available, and our coding frameworks are available in the supplementary tables. Supplementary Table S2 contains our coded data for types of intimidation experienced.

## AUTHOR CONTRIUTIONS

M.M. and K.E.R. collaborated on the idea for the project. M.M. secured funding. All authors contributed to the concept of the study and the structure of the results. P.C. and B.M. performed the literature searches. P.C., B.C., M.M., N.P. and K.E.R. coded the data. P.C., B.M. and K.E.R. conducted the data analysis. K.E.R. wrote the manuscript. All authors reviewed and approved drafts.
