# Supplementary material for: Intimidation against advocates and researchers in the tobacco, alcohol and ultra-processed food spaces: a review
Source: Health Promot Int. 2024 Nov 21;39(6):daae153. doi: 10.1093/heapro/daae153 (PMC11579607; doi:10.1093/heapro/daae153)
Supplement: daae153_suppl_Supplementary_Files_1 [file daae153_suppl_supplementary_files_1.docx]

Supplementary File 1. Adjusted forms of intimidation framework.

| Form of intimidation | Definition |
| --- | --- |
| Public discreditation – social media | An individual/ organisation **or their work** is publicly attacked, threatened, undermined or ridiculed on social media. |
| Public discreditation – traditional media | An individual/ organisation **or their work** is publicly attacked, threatened, undermined or ridiculed in print media, radio or on television. |
| Public discreditation – other | An individual/ organisation **or their work** is publicly attacked, threatened, undermined or ridiculed through other channels, e.g., publicly available consultation responses, policy hearings etc. |
| Legal threats or attacks | An individual/ organisation receives a threat of legal action against them, for example, in a letter, or is subject to legal actions. |
| Cyberattacks | An individual/ organisation experiences cyberattacks, for example, involving malware, phishing or spoofing. |
| Physical violence/intimidation | An individual experiences physical intimidation or violence or being threatened with violence (e.g., being physically attacked or given a threat of a future attack). |
| Theft/Burglary | An individual/ organisation is attacked through thefts (e.g., stealing of equipment) or burglary (e.g., break-in of offices). |
| Spying/ Surveillance* | An individual/ organisation experiences surveillance E.g. being followed, monitored online (but not hacked), going through bins. |
| Complaints to the individual/ employer* | An individual or the individual’s employer receives a complaint about the individual’s activities or work. |
| Complaints to authorities* | An authority receives a complaint about an individual or organisations. |
| Bribery** | An individual/organisation is offered money to desist with their work. |
| Other | Types of intimidation that cannot be classified in the above framework and do not feature frequently enough or provide enough information to warrant their own categories. |
| NOT USED IN CURRENT PAPER |  |
| Non-anonymous intimidating messages*** | An individual/ organisation receives an intimidating message which is, for example, a direct message on social media, a letter or phone call and the sender/caller is known. Unlike “public discretion”, here the message is not public but directly send to the individual/ organisation. |
| Anonymous intimidating messages*** | This is very similar to “Non-anonymous intimidating messages” with the only difference being that here the sender/ caller is not known. |
| Disseminating false information about individual/organisation among policymakers/ in hearings**** | False information about an individual/ organisation is shared. |

Note: *Most of the ‘other’ examples from the previous framework were turned into their own categories in a subsequent paper by Matthes et al (2023) (Matthes BK, Alebshehy R, Gilmore AB. “They try to suppress us, but we should be louder”: a qualitative exploration of intimidation in tobacco control. Globalization and Health. 2023;19(1):88.) and our paper supports their inclusion as standalone categories. **The current work supported the inclusion of a new category – bribery. ***These categories were not used as all in this paper as the intimidations found could be categorised more clearly by the other intimidation types. **** Subsumed within ‘public discreditation - other’ as policy hearings and government consultations are publicly available.
