# Supplementary material for: Intimidation against advocates and researchers in the tobacco, alcohol and ultra-processed food spaces: a review
Source: Health Promot Int. 2024 Nov 21;39(6):daae153. doi: 10.1093/heapro/daae153 (PMC11579607; doi:10.1093/heapro/daae153)
Supplement: daae153_suppl_Supplementary_Files_2 [file daae153_suppl_supplementary_files_2.docx]

Supplementary File 2. Quotes from sources which led to their inclusion in each of the intimidation types.

| Type of intimidation | Illustrative extracts from sources | Peer-reviewed references | Grey literature references |
| --- | --- | --- | --- |
|  |  |  |  |
| Public discreditation in/via traditional media (print, radio, television) | ALCOHOL  South Africa: "…certain actors who were opposed to the draft bill and a ban on alcohol advertising also appeared to use a range of personal and professional attacks to intimidate, discredit and demobilize actors who were in favour of the draft bill and the ban on alcohol advertising. The evidence for this includes: An opinion letter that appeared in a major daily newspaper, with the aim of undermining the credibility of a public health researcher (July 2011); The owner of a media and communications company placed an advert in a Sunday newspaper attacking the research and credibility of a health promotion advocate, on the grounds that she was a Muslim and therefore had ulterior motives (September 2012); The same media and communications company owner took out another advert, this time questioning the motives of the funders of a public health advocacy organisation (January 2013)." (grey lit; RESYST, 2019)  SSB  India: "The MNCs [Multi-National Corporations] (Coca-Cola & Pepsi) also tried to up the ante by casting aspersions on the integrity of CSE [Centre for Science and Environment], accusing it in effect of “untrustworthiness." (peer-reviewed lit; Vedwan, 2007)  FOOD  Philippines: "In November 2006, it [Pharmaceutical and Healthcare Association of the Philippines (PHAP)] advertised in leading newspapers stating its support for breastfeeding as best for infants, but implied that breastfeeding advocates were limiting mother's freedom of choice.." (peer-reviewed lit; Baker, 2021)  USA: "The Sugar Association dusted off what has become its stock response: The Lustig paper, it said, “lacks the scientific evidence or consensus” to support its claims, and its authors were irresponsible not to point out that the full body of science “is inconclusive at best.”… The association promptly produced an ad for newspapers and magazines exclaiming “Sugar is Safe!” It “does not cause death-dealing diseases,” the ad declared, and “there is no substantiated scientific evidence indicating that sugar causes diabetes, heart disease or any other malady…The next time you hear a promoter attacking sugar, beware the rip off. Remember he can’t substantiate his charges. Ask yourself what he’s promoting or what he is seeking to cover up. If you get a chance, ask him about the GRAS Review Report. Odds are you won’t get an answer. Nothing stings a nutritional liar like scientific facts.”" (grey lit; Taubes, 2012)  USA: "I had published an op-ed critical of Burger King. The ink was barely dry on the newspaper when I got a letter from CCF requesting the financial records for my nonprofit. It was just me and a part-time assistant. I didn't have a big staff. I had to make a lot of phone calls. I knew some other groups who had also been harassed by Berman. Because my nonprofit was so small, I didn't have to file with the IRS. I wrote back saying that I have no 990, so leave me alone. They wrote me back again saying that wasn't good enough. So then I sent them a copy of the IRS form that said I don't have to file a 990. They were obviously just trying to intimidate me and waste my time." (grey lit; Heisel, 2011)  USA: "…the site has made pictorial fun of the fact that Professor Brownell, who writes on obesity and advocates junk food taxes, is not quite as buff as a leading “food cop” is supposed to be." (grey lit; Pollan, 2006)  USA: "Within the essay, the CCF [Centre for Consumer Freedom] names specific public health advocates such as Kelly Brownell, Michael Jacobson, Marion Nestle, and John Banzhaf as a cadre of compatriots working to limit consumer choice and to erode personal responsibility. It writes that ‘‘indeed, it’s hard to find a card-carrying member of the Gastronomical Gestapo who doesn’t dismiss the concept of personal responsibility out of hand’’. The essay goes on to contend that if public health advocates have their way, government will ‘‘enact laws that would allow a waiter to decide if a patron could have dessert, much like a bartender can decide whether to pour a customer another drink. Just as cigar bars were banned in California . . . ice cream parlors could get the ax.’’ In this case, the articulation of personal responsibility to consumer freedom of choice is drawn against a clear antagonism of the variously vilified ‘‘Gastronomical Gestapo’’." (peer-reviewed lit; Thomson, 2009)  USA: "In June of 2003, Brownell spoke to the first annual ‘‘Legal Approaches to the Obesity Epidemic’’ conference and was photographed by the Center for Consumer Freedom. An intentionally unflattering picture of Brownell soon appeared on the front page of the CCF Web site, beside the large-print caption ‘‘Would you take dietary advice from this man?’’ (CCF, 2003). The CCF used an attack against Brownell’s body to discredit his authority, stating in the accompanying article: ‘‘Given Big Brother Brownell’s apparent obesity and ‘do as I say, not as I do’ attitude, this concession may just be Brownell trying to cover his largely asinine views’’ (‘‘Would You Take Dietary’’). The term ‘‘asinine’’ derives from the Latin ‘‘asininus’’ meaning ‘‘like an ass.’’ It is no coincidence that the term ‘‘ass’’ is frequently used to deprecate a corpulent individual in the pejorative slang ‘‘fat-ass.’’ Brownell’s views are ‘‘asinine’’ because he is a ‘‘fat ass’’ and therefore should not be heard, so the logic goes." (peer-reviewed lit; Thomson, 2009)  TOBACCO  Germany: "The tobacco industry, by way of representatives or medical journals, responded by framing the work of Ferdinand Schmidt as peculiar. The industry’s attacks continued into the 1990s and were successful at marginalising Schmidt in the eyes of government health administrators." (peer-reviewed lit; Bornhäuser, 2006)  Germany: "Libertarian pro-tobacco activists started using “Nazi” rhetoric to discredit journalists and public health experts. Analogies with Nazi symbols, including the use of **the yellow Star of David on a pro-smoking T-shirt** and in a TV news broadcast, were used to liken the treatment of smokers to the stigmatisation and discrimination of Jews under the Nazis." (peer-reviewed lit; Schneider, 2008)  Global: "Tobacco control activists were the persecutors. One FOREST supporter called smoke-free policies a ‘new apartheid’ and smokers the ‘victims of health fascist pass laws.’ Hen-Ry (Denmark) characterized tobacco control organizations as ‘aggressive and dominant,’ invoking a ‘smoker’s inquisition.’ The Greek spokesman said ‘fanatical’ and ‘intolerant’ tobacco control advocates believed they had a ‘right and a duty’ to impose their ideas on others, while the Swedish spokesman referred to ‘Ayatollahs of antismokers’." (peer-reviewed lit; Smith, 2006)  Thailand: "ECST [ENDS Cigarette Smoking Thailand] employed standard industry tactics in efforts to discredit tobacco control researchers and advocates and undermine policy relevant research: criticise tobacco control advocates in the press, attack advocates on its Facebook page… use third parties, including physicians and researchers to attack tobacco control advocates’ claims. For example, ECST launched a campaign ‘We don’t accept TRC’ on Change. org to discredit TRC and oppose it leading a study on the appropriate ENDS control policy for Thailand." (peer-reviewed lit; Patanavanich, 2021)  USA: "The tobacco industry successfully muzzled this revelation and continued to maintain to the general public that Hirayama’s work was flawed. The cover ups and prevention of research with the potential to produce unfavourable results continued. In 1990, Dr. Adlkofer in Munich proposed a plan for a lifetime animal inhalation study of ETS that was successfully quashed." (peer-reviewed lit; Drope, 2001)  USA: "Canadian John Luik wrote 'Pandora's box: The dangers of politically corrupted science for democratic public policy' to attack the recently released EPA report that had classified ETS as a human carcinogen". (peer-reviewed lit; Drope, 2001)  USA: "Bait anti-tobacco forces to criticize industry efforts. Focus media on anti’s extremism. Anticipate and blunt antis strongest points . . . for positioning purposes." (peer-reviewed lit; Landman, 2002)  USA: "On 18 November 2001, David Phelps and Deborah Caufield Ryback, business reporters from the Minneapolis Star-Tribune, published an extensive front page article criticising MPAAT [Minnesota Partnership for Action Against Tobacco]. This story, the first of 13 that would run through April 2002, alleged that MPAAT had ignored its charter by concentrating on community norm change, particularly promoting smokefree environments, rather than funding individual smoking prevention and cessation…. Phelps and Caulfield-Ryback’s stories also included accusations of misconduct in the evaluation and funding of grants and assertions of conflicts of interest…" (peer-reviewed lit; Ibrahim, 2004)  USA: "The director of smoking and health for tobacco company RJ Reynolds sent a letter to the editor of many newspapers calling Glantz a ‘‘back door prohibitionist’’ who was ‘‘scaring the American public with outlandish claims that are simply not supported by scientific fact.’’ (RJ Reynolds’ public relations agency, Hill & Knowlton, had found the ‘‘back door prohibition’’ message to be most effective with the public.) After 1991, the industry moved from direct criticism to using third parties. " (peer-reviewed lit; Landman, 2009)  USA: "Philip Morris proposed to alter what it perceived as a troublesome scientific environment by ‘‘raising the issue of [scientists’] credibility and their integrity.’’ The plan, titled ‘‘Action Plan: Scientists,’’ proposed ‘‘exposing .. . scientists who engage in shoddy research to their peers and in the media,’’ and it noted that ‘‘scientists are very concerned about the opinions their peers hold of them and their research.’ The plan proposed ‘‘elevat[ing] the issue of public funding (primarily federal) to conduct anti-tobacco ... research’’ and using third parties to run advertisements attacking scientists." (peer-reviewed lit; Landman, 2009) | Baker, 2021; Bornhäuser, 2006; Drope, 2001; Ibrahim, 2004; Landman, 2002; Landman, 2009;  Patanavanich, 2021; Schneider, 2008; Smith, 2006; Thomson, 2009; Vedwan, 2007 | Heisel, 2011; Pollan, 2006; RESYST, 2019; Taubes, 2012 |
| Public discreditation on social media | ALCOHOL  New Zealand: “’If there was ever a case of demonstrating once and for all the Professor Doug Sellman is mad, this article “Drunks steal sanitiser for alcohol” proves it…any ounce of credibility that this guy once had has long-since evaporated…Maybe it’s time Doug has a quiet beer under a tree and learns to calm the f**k down.” Graham [industry consultant] had been paid to sit in his chic Parnell offices writing this stuff and then to pay Slater [owner, right-wing blog ] to publish it.” (grey lit; Hager, 2014)  SSB  New Zealand: "Rather than addressing the health problems associated with sweet drinks, they focused the response on attacking a man named Tony Falkenstein, who was the New Zealand contact on the class action advertisement…’Follow up piece coming shortly for tomorrow afternoon. 3 hits smashing him good and proper’." (grey lit; Hager, 2014)  USA: "Marion Nestle, Food Fascist. Sound harsh? After our latest check-in with everyone's favorite anti-pleasure nutritionist, we think it's completely appropriate." (grey lit; CCF, 2010)  FOOD  New Zealand: "There were…seven Whale Oil [right-wing blog in receipt of industry funding] posts in a row in 2012 attacking ‘The Breastapo’, people advocating and supporting breast feeding of babies, and objecting to regulations on the sale of infant formula. Like the tobacco ‘hits’, the blog posts contained specific information on government funding (‘troughing’ from the ‘public trough’) Received by groups such as Women’s Health Action, which Graham had requested from the ministry a few months before these posts." (grey lit; Hager, 2014)  USA: "The latest anti-food jihad from the Center for Science in the Public Interest — an effort to “ban 2 percent and whole milk from schools” — has been labeled “laughable” by the Wisconsin State Journal. If the Journal thinks this latest effort to steer kids from a vital source of calcium is ridiculous, it should look closely at CSPI’s other attacks against foods it doesn’t like." (grey lit; CCF, 2004)  USA: (in response to chain restaurants having to label the nutritional information on their meals) "Tell it to the Center for Science in the Public Interest, which zealously promotes the unworkable labels, and John “Sue the Bastards” Banzhaf, who would be first in line to cash in when your steak isn’t cut — or your ice cream isn’t scooped — exactly the same as the next guy’s." (grey lit; CCF, 2004)  TOBACCO  Global: "Personal abuse and four-letter invective in blogs, tweets and emails are commonplace. Among the gentler comments, tobacco control leaders are liars, frauds, imbeciles, stupid, hate-filled, unethical, dishonest, hysterical, contemptible, insanely wicked, evil, sick, lunatics and paid tools of the pharmaceutical industry…My integrity was recently impugned by a micro-organisation with a lofty sounding name, whose website showed precisely one blogger, and which is associated with groups with a history of tobacco funding. They made imputations that were nasty and untrue — but attracted media coverage, which was presumably the intention. All that is, of course, over and above the abuse we get from those with commercial interests in tobacco and alcohol and groups directly associated with them, such as the Australian Hotels Association, who specialise in meaningless phrases such as “wowser”, “nanny state”, “zealot” and “prohibitionist”, and attack health groups including the Australian Medical Association, or prominent individuals such as police commissioners, with wild assertions and distasteful comments about personal motivation." (grey lit; Daube, 2015)  New Zealand: "There was another pattern too. In addition to the paid posts, there was a Whale Oil commenter called ‘Naylor’ who commented on the funding for Te Reo Marama and other tobacco-related subjects…In fact it was Carrick Graham [Industry consultant]. He used the Whale Oil comments to say [derogatory] things a smooth PR consultant would never say under his own name." (grey lit; Hager, 2014)  New Zealand: “At the High Court in Auckland on Wednesday Graham [industry funded blogger] admitted the statements made against the men [public health advocates] were "untrue, unfair, offensive, insulting and defamatory" and were done to "advance the interests of industry".” (grey lit; Espiner, 2021)  Thailand: “ECST [ENDS Cigarette Smoking Thailand] employed standard industry tactics in efforts to discredit tobacco control researchers and advocates and undermine policy relevant research: criticise tobacco control advocates … on its Facebook page”….” (peer-reviewed lit; Patanavanich 2021) | Patanavanich, 2021 | CCF, 2004; 2010; Daube, 2015; Espiner, 2021; Hager, 2014 |
| Public discreditation through other channels/ platforms (e.g., public statements | ALCOHOL  Australia: "The second of these facts is usually ignored in the advocacy of population level measures, particularly by NAAA members who are often paid advisers to government on alcohol policy" and "… though the National Drug Research Institute is a very active member of the extreme anti-industry NAAA group." (peer-reviewed lit; Avery, 2016)  Australia: "Shane Tremble who falsely claimed there ‘is no such evidence’ showing that the size of a liquor outlet is related to alcohol harm…This is not the first occasion on which Woolworths has attempted to denigrate scientists’ independent peer-reviewed research." (grey lit; FARE, 2017)  Malawi: "During these meetings [between government and stakeholders], alcohol industry representatives were reported to have facts that countered what government and NGOs were presenting, specifically highlighting that alcohol is not as harmful as the NGOs were stating. Some key informants also suggested that the alcohol industry was reported to have approached some members involved in the alcohol policy formulation to influence them to support the views and perspectives of the industry." (peer-reviewed lit; Matanje Mwagomba, 2018)  FOOD  USA: "As a discrediting mechanism, Nestle promoted the idea that its changes of policy were not being “recognized” by activist leadership, advancing the notion that intransigent, “ideological” leadership of the boycott was at odds with “reasonable, humanely committed” boycott supporters." (peer-reviewed lit; Johnson, 2020)  Brazil: "We have done an analysis of who was criticising the concepts, that classification of foods and most of these people were related to the food industry so it was not actually the food industry but the food industry had connections with certain people from the US, from Brazil, from France, whose finances were quite close to the industry - they were criticising the work of colleagues working on the NOVA classification. So it was not specifically one individual but it was questioning the work of groups of individuals on the NOVA classification." (grey lit; Mialon, 2021)  "Also [they] said that those working on food regulation were described as "too radical"." (grey lit; Mialon, 2021)  TOBACCO  Australia: "AHA [Australian Hotels Association of Western Australia] directly undermined individual independent public health researchers " (peer-reviewed lit; Avery, 2016)  Finland: "A clinical chemist wrote a comprehensive report for Amer-Tupakka, attacking studies showing hazards caused by ETS. An internal memo considered how the doctor in question could most credibly be presented as an independent critic of the hazards caused by ETS [Environmental Tobacco Smoke]." (peer-reviewed lit; Hiilamo, 2003)  Hong Kong: "‘We must attack the anti-smoking… Zealots’’ Having identified and monitored known tobacco control groups, the industry then sought to ’’carefully target our opponents’’ by the demons of overzealous moral righteousness" (peer-reviewed lit; Knight, 2004)  Ukraine: "Opponents also used media advocacy to discredit tobacco control proponents, suggesting that these proponents are only concerned about receiving foreign funding rather than improving public health in Ukraine." (peer-reviewed lit: Hoe, 2021)  USA: "Other strategies the industry used or contemplated using against STAT/INFACT included publicly… attempting to portray the organizations’ leaders as too “extreme”." (peer-reviewed lit; Malone, 2002)  USA: "A third major purpose [of flooding the ASSIST programme with FOI requests] was to expose alleged waste and inefficiency in ASSIST programs, conflicts of interest, and lack of compliance with rules. A fourth was to use information obtained to develop counterproposals." (peer-reviewed lit; White, 2004)  USA: “Philip Morris created a plan to rehabilitate its image and promote its business interests by developing "new strategies to isolate tobacco control advocates, showcasing their “extremism” and using it against them, as in PM's earlier “Project Sunrise”.” (peer-reviewed lit; Yang, 2008)  “Islamic Countries”  “Countries with predominantly Islamic faith population: Transnational tobacco companies sought to counter growing Islamic opposition to tobacco use by depicting supporters as religious extremists and, by extension, tobacco control as another form of extremism...fundamentalism.” A 1982 letter observes, “In the more fundamentalist groups . . . smoking is actively discouraged and is frequently the subject of the Friday sermon.” Similarly, a 1983 report on Nigeria observes that “only the discredited ‘fundamentalists’ have attempted to generate antismoking.” BAT visitors to Saudi Arabia in 1991 also criticized “the religious” (peer-reviewed lit; Petticrew, 2015) | Avery, 2016; Hiilamo, 2003; Hoe, 2021; Johnson, 2020; Knight, 2004; Matanje Mwagomba, 2018; Petticrew, 2015; White, 2004; Yang, 2008; | FARE, 2017; Mialon, 2021 |
| Legal threats/action | SSB  Brazil: "I first received a couple of offers from Coca Cola to become friends with them and to participate in some calls for proposals that they were having about agroecology and wanted to network and to have conversations, and well 'no thanks, I'm not willing to do that' and then we kept on doing our regular work of exposing the risks of sugary drinks and so on and then we had the Brazilian nutrition conference and then we did like this huge, inspired by Pepsi Co, this huge inflatable can there with diabetes instead of Coca Cola and then I receive an intimidation letter immediately after... it was just an intimidation, like, stop doing that or continue with that we're gonna sue." (grey lit; Mialon, 2021)  Colombia: "The backlash was fierce. A Colombian Government agency, responding to a complaint by the nation’s leading soda company that called the ad misleading, ordered it off the air. Then the agency went further: It prohibited Dr Cerón and her colleagues from publicly discussing the health risks of sugar, under penalty of a $250,000 fine." (grey lit; Jacobs, 2017)  India: “…there was a shift in the strategy when The Coca Cola Company and PepsiCo quietly decided to drop their libel suit against CSE, an action the CEO of PepsiCo justified as a step to reduce the risk of harm to their brand equity. “Taking CSE to court won’t improve our brand image”. (peer-reviewed lit; Vedwan, 2007)  FOOD  Fiji: "We were threatened [a few years ago]. Right at the eleventh hour, the day before we were supposed to go and present [our work], we started receiving faxes from some of the biggest and richest law firms in Fiji, who represent some of the big companies...they actually threatened 'if this goes public, we will [sue you]. We had to withdraw everything." (grey lit; Mialon, 2021)  Kenya: "In June 1974, a Swiss organization called the Arbeitsgruppe Dritte Welt (Third World Working Group) translated the report into German and altered its title to Nestlé tötet Babies (Nestlé Kills Babies). In response, Nestlé sued the group in Bern for libel… Nestlé won the libel case…". (peer-reviewed lit; Sasson, 2016)  Nigeria: "Nestle sued the group [Swiss Berne Third World Action Group] for libel". (peer-reviewed lit; Anaemene, 2013)  TOBACCO  Brazil: "…we have been sued by the tobacco industry in the past because of [a] 30 seconds advertisement that we had about tobacco point-of-sale advertisement of tobacco, and that was a real lawsuit." (grey lit; Mialon, 2021)  Finland: "Since 1988, tobacco companies have been defendants in lawsuits in Finland. In this position they accused the plaintiff of exploiting the trials. The companies claimed personal compensation for legal expenses from the plaintiff’s attorneys. In the judgement given by the Helsinki Court of Appeal at the end of 1998, the plaintiff’s attorneys were, exceptionally, made personally liable for legal expenses." (peer-reviewed lit; Hiilamo, 2003)  Germany: “Members of the German subsidiary of the US smokers’ rights organisation Fight Ordinances and Restrictions to Control and Eliminate Smoking (FORCES), Netzwerk Rauchen—Forces Germany eV, discussed suing a German tobacco control champion and head of the German WHO Collaborating Center for Tobacco Control, alleging “Volksverhetzung” (Agitation of the People), an accusation typically directed against neo-Nazis. Under German law incitement of hatred against a minority is punishable with up to five years in prison.” (peer-reviewed lit; Schneider, 2008)  India: "The respondents stated that legal challenges (litigations, RTIs etc.), offering undue favors, issuing threats are being used as weapons by the TI to interfere in the implementation of tobacco control laws."(peer-reviewed lit; Goel, 2021)  USA: "On 19 April 2002, Hatch [Minnesota District Attorney] filed a motion with the court seeking to dissolve MPAAT and move its funds to the Minnesota Department of Health and the University of Minnesota. This action would bring the tobacco control programme under the control of the legislature and governor, the precise situation that MPAAT was designed to avoid. On 1 May Hatch further proposed replacing the MPAAT board with a five-person committee, two of whom were former governors Arne Carlson and Wendell Anderson. Carlson had accepted $5000 in campaign contributions from the tobacco industry between 1989 and 1994, used three of the tobacco industry’s top lobbyists in Minnesota for his re-election campaign, and maintained a pro-tobacco position on legislation." (peer-reviewed lit; Ibrahim, 2004)  USA: "In addition to threatening to withhold payment, Lorillard altered its claim for damages in the pending case against Legacy from $1 to the return of MSA [Master Settlement Agreement] payments made by the company since 1999 to the escrow account set up in the MSA. While Lorillard quickly reversed its decision regarding the annual payments under pressure from NAAG and made the 31 March 2003 MSA payment (to both the states and Legacy), the company maintained its request for damages equal to all MSA payments made by the company. If successful and used as a precedent by other tobacco companies who were signatories to the MSA, this action would lead to the end of the American Legacy Foundation." (peer-reviewed lit; Ibrahim, 2006)  USA: "The National Smokers’ Alliance, a smokers’ rights organisation created for PM [Philip Morris] by the public relations firm Burson Marsteller, sued Roswell Park Cancer Institute in 1999 for allegedly violating a federal lobbying law." (peer-reviewed lit; McDaniel, 2006)  USA: "The tobacco industry used a number of tactics to disrupt ASSIST. These included **litigation**, formal complaints, audits, and exhaustive FOIA requests. A task force convened by Philip Morris in 1993 identified the main objective for these activities: “Launch investigation of ASSIST program in Congress and in state legislatures. . .. Use overall investigation to develop a public relations program designed to erode credibility of opponents over the long term." (peer-reviewed lit; White, 2004)  USA: "On July 1, 1997, Californians for Scientific Integrity sued the University of California, the California Department of Health Services, and the California Department of Education in California Superior Court, charging Glantz with engaging in scientific misconduct at taxpayer expense by intentionally misrepresenting the data in his 1994 restaurant study… On November 20, 1997, the Court dismissed the suit on the grounds that there was no legal basis for a claim against the University…"(peer-reviewed lit; Landman, 2009) | Anaemene, 2013; Bialous, 2001; Hiilamo, 2003; Goel, 2021; Ibrahim, 2004; Ibrahim, 2006; Landman, 2009; McDaniel, 2006; Sasson, 2016; Schneider, 2008; Vedwan, 2007; White, 2004; | Jacobs, 2017; Mialon, 2021 |
| Complaints to government bodies/international organisations/national and international authorities | FOOD  USA/Global: "[T]he Sugar Association, enlisted the support of officials high in the US government and led a vigorous attack on both the report and the WHO. Beginning with letters to the WHO's director general, the Sugar Association criticised both the science and the process by which the report was prepared and asked that it be stopped, or at the very least, delayed. Not receiving the desired response, the industry quickly raised the stakes when two US senators, Larry Craig and John Breaux, co-chairs of the US Senate Sweetener Caucus implored Thompson to use his 'personal intervention' in blocking the report. Thompson attempted to deliver. A DHHS assistant secretary sent a twenty-eight-page, single-spaced report to the WHO picking at the science and making the same three points promoted by the industry: personal responsibility should be the emphasis; there should be a stronger focus on physical activity; and there are no good and bad foods. Thompson dispatched this assistant to Geneva at the time of a key WHO meeting, pressuring constituent countries to block the report. The Sugar Association simultaneously played its ultimate card. Expressing concern for “the hard working sugar growers and their families,” its president again wrote the WHO, vowing to use “every avenue possible to expose the dubious nature” of the report, “including asking Congressional appropriators to challenge future funding of the U.S.’s $406 million contributions to the WHO.” This is the WHO that deals with AIDS, malnutrition, infectious disease, bioterrorism, and more, threatened because of its stance on sugar."(peer-reviewed lit; Brownell, 2009)  SSB  Colombia: "The backlash was fierce. A Colombian Government agency, responding to a complaint by the nation’s leading soda company that called the ad misleading, ordered it off the air."(grey lit; Jacobs, 2017)  TOBACCO  Netherlands: “On 3 February 2011, the industry [via the Dutch employer organisation VNO-NCW] sent a letter to the ministry complaining about advocacy organisations’ use of Article 5.3 and sought reassurance that industry–government exchanges would remain unaffected.” (peer-reviewed lit; Willemsen, 2019)  Nigeria: "Key informants reported that the industry may also have been working quietly to destabilise civil society groups through the introduction of bills to regulate the activities of non-governmental organisations and may have promoted two weaker tobacco control bills in both houses of the National Assembly between 2011 and 2013." (peer-reviewed lit; Egbe, 2019)  Thailand: "In 2015, the Thai government had accepted and acted on a proposal from the Thai Tobacco Monopoly (TTM) to investigate and reorganize [the] Thai Health Foundation." (peer-reviewed lit; Assunta, 2017)  Thailand: "ECST [ENDS Cigarette Smoking Thailand] employed standard industry tactics in efforts to discredit tobacco control researchers and advocates and undermine policy relevant research…[including] submit complaint letters against tobacco control researchers to government agencies…" (peer-reviewed lit; Patanavanich, 2021)  USA: "[A] complaint filed by the Minnesota Grocers Association with the Minnesota Ethical Practices Board and to a letter to the state auditor alleging unlawful use of federal taxpayer dollars in October 1995. The complaint accused 16 groups that were ASSIST grantees of misusing ASSIST funds by encouraging stronger tobacco control laws and of violating the state’s lobbying disclosure rules." (peer-reviewed lit; Bialous, 2001)  USA: “…the Tobacco Institute concluded in 1994 that: "The best way to use the new lobbying prohibition may be to bring a complaint to the Inspector General of Health and Human Services. . . . The complaints coupled with political pressure from the Hill eventually might be more effective than a FAR [Federal Acquisition Regulations] complaint to the GAO [General Accounting Office]. . . . By sending the complaints to the IG [inspector general], we could coordinate a grassroots effort that would send dozens of complaints to the IG’s office, forcing the IG to address the problem. . . . If the IG dismisses the complaints . . . his actions will be of interest to the appropriate congressional oversight committees. And, given recent political changes, HHS [Health and Human Services] may be more sensitive to congressional pressure.”” (peer-reviewed lit; White, 2004)  USA: "In 1996, Representative Harold Rogers (Republican, Kentucky) urged the Internal Revenue Service to investigate CTFK’s tax exempt status; that same year, the Department of Health and Human Services’ Inspector General audited the American Stop Smoking Intervention Study (ASSIST) at the request of the Appropriations Subcommittee, chaired by tobacco industry allies Representatives Henry Bonilla (Republican, Texas) and Ernest Istook (Republican, Oklahoma)). PM also planned to ‘‘identify opportunities to tighten federal and state funding and lobbying requirements that would directly impact the ATI…. In 1997, a draft letter from North Carolina representative Walter Jones, found in the PM documents, urged the Internal Revenue Service to investigate the American Heart and Lung Associations’ political activity; his office provided PM with versions of the letters he sent requesting an investigation." (peer-reviewed lit; McDaniel, 2006) | Assunta, 2017; Bialous, 2001; Brownell, 2009; Egbe, 2019; McDaniel, 2006; Patanavanich, 2021; White, 2004; Willemsen, 2019; | Jacobs, 2017 |
| Complaints to the individual or organisation | FOOD  Colombia: "[T]he group of defenders suffered great pressure not to participate in confronting the shareholders, who expressly requested that Esperanza not speak." (grey lit; Lorenzo, 2019)  Philippines: “A US-based lobby group for the baby food industry, wrote a letter to UNICEF’s Director General Ann Veneman…claimed the breastfeeding promotion activities of UNICEF’s Philippines country office ‘misrepresents the available scientific evidence regarding the alleged risks of not breastfeeding. A letter was also sent to the UNICEF regional office in Bangkok, complaining about the ‘unscientific’ remarks and questioning the competency of the UNICEF-Philippines country representative. A similar complain was made against the WHO country representative.” (peer-reviewed lit; Baker, 201)  USA: “I received a letter from The Salt Institute’s lawyers challenging our data and demanding the whole data file so that further and “proper” analyses could be done. That could be very intimidating, you know. In fact, many people prefer not to get involved in the public arena because they don’t want all those hassles, including the time it consumes. It consumes a lot of time.” (grey lit; Freudenberg, 2007)  USA: “I had published an op-ed critical of Burger King. The ink was barely dry on the newspaper when I got a letter from CCF requesting the financial records for my nonprofit. It was just me and a part-time assistant. I didn’t have a big staff. I had to make a lot of phone calls. I knew some other groups who had also been harassed by Berman. Because my nonprofit was so small, I didn’t have to file with the IRS. I wrote back saying that I have no 990, so leave me alone. They wrote me back again saying that wasn’t good enough. So then I sent them a copy of the IRS form that said I don’t have to file a 990. They were obviously just trying to intimidate me and waste my time.” (grey lit; Heisel, 2011)  UNKNOWN SECTOR/COUNTRY  Unknown country: “[An] email to complain about the work…”" (grey lit; Mialon, 2021) | Baker, 2021 | Freudenberg, 2007; Heisel, 2011; Lorenzo, 2019; Mialon, 2021 |
| Surveillance | FOOD  Switzerland: "In 2003, a group of activists with the Association pour la Taxation des Transactions pour l'Aide aux Citoyens (ATTAC) in Vaud, Switzerland, started working on a book on the global policies of Nestlé. A Securitas employee infiltrated the group under a false name (Sara Meyland) in order to attend the ATTAC meetings about the planned book. In June 2008, Temps Présent, a Swiss TV program, revealed that the Securitas agent had briefed Nestlé security personnel as well as corporate communications staff about the meetings that she attended including ones held in private homes." (grey lit; Chatterjee, 2013)  SSB  Australia: "Coke sent employees to take notes are Marion Nestle’s Sydney Australia talk on the soda industry. Dr. Nestle of New York University is the author of Soda Politics and an outspoken critic of the soda industry." (grey lit; Huehnergarth, 2016)  Australia: "The hacked emails included a January 2016 message from the director of an Australian agency doing public relations for Coca-Cola with notes taken at a lecture I had just presented to the Sydney chapter of the Nutrition Society of Australia. The emailed notes on my lecture — quite nicely done, actually — name some of the people attending my talk, review its content, and advise Coca-Cola to monitor my future presentations, research, and presence on social media”. (grey lit; Nestle, 2018)  Colombia: "It began with menacing phone calls, strange malfunctions of the office computers, and men in parked cars photographing the entrance to the small consumer advocacy group’s offices." (grey lit; Jacobs, 2017)  Mexico: See cyberattacks (grey lit; Jacobs, 2017)  TOBACCO  Asia: "Over the following two decades, the industry built up a comprehensive dossier on its opponents including those in the Asian region. During this time they sought to know the anti-smoking movement’s strategies and objectives. PM, for example, was clear on the tobacco control’s objectives and strategies identifying their opponents’ strengths (‘‘easy access—government and media’’) and vulnerabilities (‘‘strident’’)." (peer-reviewed lit; Knight, 2004)  Australia: "“The flip side of destroying our own documents was to try to acquire documents held by the anti-smoking organisations.” How did TIA do that? “As the anti-smoking organisations were not as careful as the industry about getting rid of documents, the TIA paid individuals to go through the dumpsters and rubbish of antismoking organisations in order to find any potentially relevant documents relating to their plans or their funding.” TIA employees themselves did not go through the anti-smoking trash, but contracted with a private inquiry agent, Winston Gregory & Associates, who employed a number of individuals who would go through the anti-smoking organisations’ garbage.". (grey lit; Simpson, 2005)  USA: "Beth Lancaster of Fleishman Hillard and R.J. Reynolds consultant Susan Heenan attended STAT’s 1992 meeting, according to the STAT participants list found among other Tobacco Institute documents. Lancaster (and possibly others) secretly tape-recorded the sessions, despite careful and explicitly announced security measures by the conference organizers. " (peer-reviewed lit; Malone, 2002)  USA: "…tobacco industry surveillance extends beyond attendance at public meetings. The industry response in the case described above included aggressive intelligence gathering, use of public relations specialists as spies, and covert audiotaping. In addition, the documents show that cigarette companies coordinated among themselves to share information and maintained detailed lists of industry critics." (peer-reviewed lit; Malone, 2002)  USA: “…one example of outright infiltration by tobacco industry operatives occurred in Colorado in 1992, where an individual apparently working for the Denver public relations firm of Karsh & Hagan reported on a meeting of an ASSIST coalition in Fort Collins: ‘*I arrived after the meeting commenced and despite my effort to remain invisible, ended up seated at the head of the table. I signed in as a student and hoped that my baggy clothes and backpack would make this credible. . . . Would advise future “plants” to arrive late and leave early, avoiding the awkward small talk with other attendees that might create suspicion*’." (peer-reviewed lit; White, 2004)  USA: "From 1997 to 1999, PM consultants conducted extensive research on tobacco control organisations, including their mission, leadership, funding sources, tax status, membership, and priorities, and federal and state tobacco control activities. PM consultants also monitored tobacco control advertisements, press conferences, websites, list-serves, and publications. Issues management fed this information into the ‘‘Common Ground’’ database, searchable by tobacco control issues, groups, people, and television and print advertisements." (peer-reviewed lit; McDaniel, 2006)  USA: "…[M]easures involving tracking of documents, activities, and names of COMMIT activists in Utica….led to discussions at RJR about developing an “anti-smokers database,” as indicated in an internal letter: . . . we don’t want to be in a position of developing a Richard Nixonesque ‘enemies’ list… The existence of such list could, in and of itself, be a negative P.R. story... The reasons for putting together such a database are simple: one, to insure our communications don’t go directly to antis, thus making it harder for them to pull off stunts such as they did in Utica (though hardly making it impossible)… A document from Philip Morris for its 1991 stockholders meeting identifies 20 pages of “antis,” both individuals and organizations". (peer-reviewed lit; Carlini, 2006)  UNKNOWN SECTOR/ COUNTRY  Latin America: "[S]o you have small cameras, it's like a James Bond movie but we know that in some countries, they use for example [inaudible] were *inaudible* cameras and then they can take videos in public meetings of what people are saying." (grey lit; Mialon, 2021)  Unknown country: "I know of people who received phone calls telling them that they knew where their kids was [sic] and that they were following their kids after school." (grey lit; Mialon, 2021) | Carlini, 2006; Knight, 2004; Malone, 2002; McDaniel, 2006; White, 2004 | Chatterjee, 2013; Huehnergarth, 2016; Jacobs, 2017; Mialon, 2021; Nestle, 2018; Simpson, 2005; |
| Freedom of Information requests | FOOD  New Zealand: "Other health-related attacks can be directly traced to information requests Graham [industry consultant] has sent to the Ministry of Health. " (grey lit; Hager, 2014)  TOBACCO  India: The respondents stated that legal challenges (litigations, RTIs etc.), offering undue favors, issuing threats are being used as weapons by the TI [tobacco industry] to interfere in the implementation of tobacco control laws…. (peer-reviewed lit; Goel, 2021)  New Zealand: "In November 2013, for instance, he [industry consultant working for the tobacco industry] requested ‘copies of all communications, including any reports, emails or other correspondence…[and] any contracts or agreements for services’ for two women: Sue Taylor and Teresa Taylor of T&T Consulting and Te Reo Marama, who were involved in Maori smoking cessation programmes. A few months later a Whale Oil [right-wing political blog] post appeared…saying ‘This week the health Select Committee will hear again from a bunch of troughers demanding NZ truck ahead with plain packaging for ciggie packs.’ The ‘prime example’ of this was Sue Taylor of Te Reo Marama." (grey lit; Hager, 2014)  USA: "The tobacco industry has burdened media staff with massive requests for information under state and federal freedom of information acts as a means to delay or diffuse progress on reducing tobacco consumption."(peer-reviewed lit, Ibrahim, 2006)  USA: "Tobacco industry strategists agreed that a crucial first step was to gather extensive information on the ASSIST programs, primarily through “aggressive open records efforts” using the federal Freedom of Information Act (FOIA) and similar state legislation…Some of these requests were extraordinarily detailed and demanding, requiring a considerable amount of staff time to meet…Allies could send FOIA requests using sample letters provided by the tobacco companies. " (peer-reviewed lit; White, 2004)  USA: "In Minnesota, the attorney general [who had TI links] challenged grantees from the Minnesota Partnership for Action Against Tobacco (MPAAT), a foundation created by the Minnesota tobacco settlement to run a tobacco control programme, to produce information which resulted in one grantee spending $20 000; none of the material that was provided to the attorney general was ever used or seen in subsequent litigation. " (peer-reviewed lit, Ibrahim, 2006)  USA: "Progress in California’s media campaign was virtually halted for months while staff members worked to produce evidence requested by the tobacco companies" (peer-reviewed lit, Ibrahim, 2006)  USA: “A fax sent to Steven Parrish, vice president of Philip Morris USA, from the law offices of Shook, Hardy & Bacon, states: ‘*Dear Steve: In August 1991, we requested from the National Cancer Institute records pertaining to the Community Intervention Trial for Smoking Cessation (COMMIT). I believe it was John Nelson at Philip Morris who asked you for this material. NCI produced about 200 pages of documents and I sent those to you in November. Now, 13 months after our request, NCI has produced another 1,900 pages .* ..’” (peer-reviewed lit; Carlini, 2006)  USA: "[P]rogress in making new ads in California was virtually halted for months in 2002 while staff members worked to produce evidence requested by the tobacco companies in an unrelated lawsuit." (peer-reviewed lit; Ibrahim, 2007)  USA: "But Virginia’s ASSIST efforts were hampered by the tobacco industry, which used tactics such as overwhelming the ASSIST 800 number during phone banking and submitting Freedom of Information Act requests to coincide with ASSIST’s deadlines." (peer-reviewed lit; Fallin, 2015)  UNKNOWN SECTOR/ COUNTRY  Unknown country: "Asking to see for example the data from our research, the email exchange between researchers, sometimes there is no specific objective in doing that - just to delay you..."(grey lit; Mialon, 2021) | Carlini, 2006; Fallin, 2015; Goel, 2021; Ibrahim, 2006; Ibrahim, 2007; White, 2004 | Hager, 2014  Mialon, 2021 |
| Physical threats/violence | SSB  Colombia: "Then at dusk one day last December, Dr. Esperanza Cerón, the head of the organization, said she noticed two strange men on motorcycles trailing her Chevy Sedan as she headed home from work. She tried to lose them in Bogota’s rush-hour traffic, but they edged up to her car and pounded on the windows. “If you don’t keep your mouth shut, one man shouted, she recalled in a recent interview, “you know what the consequences will be." (grey lit; Jacobs, 2017)  "Dr Cerón was startled awake at 5am by a call to her cellphone. “Shut up you old wench” the caller yelled, according to a report she filed with the Fiscal General de la Nación, Colombia’s prosecutorial agency." (grey lit; Jacobs, 2017)  "Dr. Cerón was walking to the gym when a man, his face obscured by a hooded sweatshirt, accosted her with the same message. “Cállese,” he yelled before walking away, or, “keep your mouth shut”." (grey lit; Jacobs, 2017)  "[S]he received a series of threats for a campaign that her organisation, Educar Consumidores (Educate Consumers), launched to prevent the population, particularly children, from suffering the damage caused by excessive consumption of sugary drinks. In this case, the soft drink industry did not hesitate to use all possible strategies, not only putting lobbyists in the Colombian Congress but also using telephone interventions, death threats and intimidation against Esperanza and her team to silence their voices." (grey lit; Lorenzo, 2019)  TOBACCO  Argentina: "Some articles contained personal threats to tobacco control advocates and considering the history of arbitrary political repression in Argentina, these threats were to be taken seriously." (peer-reviewed lit; Mejia, 2008)  Indonesia: "In 2012, Tara Singh Bam, deputy regional director of the International Union Against Tuberculosis and Lung Disease, discovered “wanted” posters with his face and those of nine other antismoking advocates…A year later, he said, an intruder pushed into the lobby of his Jakarta apartment just as he was taking his children to school. “He grabbed my hand and said ‘You must leave my country as soon as possible,’” said Dr. Bam, who is from Nepal. “Then he blew smoke in my face. My children started crying — and he left.” And just two years ago, he said, he received a Facebook message warning: “Do not interfere in our tobacco affairs.” It ended with “Your coming made the atmosphere not good”." (grey lit; McNeil, 2018)  Nepal: "Key informants reported that in June 2010 tobacco industry representatives made death threats to tobacco control advocates and their families by telephone and SMS text messages." (peer-reviewed lit; Bhatta, 2020)  Nigeria: "…more than a dozen men with AK-47s shot their way into Akinbode Oluwafemi’s home in Lagos, Nigeria. They killed his house guard and his brother-in-law, and briefly held a muzzle to the head of one of his year-old twins...Someone called a friend of mine and said I should shut up or be killed,” he said. Two years ago, as he was interviewed on Africa Independent Television, someone called another friend and said: “Your boy is on TV again — we have arranged that he’ll be killed." (grey lit; McNeil, 2018)  UK: "One article, called "sniper the flappers," hosted by the campaign group Freedom2choose's website in March, suggested shooting staff of Action and Smoking and Health and Cancer Research UK, entrepreneur and anti-smoker Duncan Bannatyne..." (grey lit; Campbell, 2012)  UK: “Staff were subjected to abusive and threatening phone calls and emails after publishing an article in January about the illegal trade in a smokeless tobacco called snus. One caller left a message to be passed to Anna Gilmore, the group’s director, saying: "Tell her that I know people that would like to meet with her in a dark alley." The group received about seven calls a day for two months. "The ones I answered were intimidatory; the tone was aggressive. Many of my team felt threatened and worried by this.”" (grey lit; Campbell, 2012)  UK: “Linda Bauld of Stirling University called the police last September when a pro-smoking blogger calling himself Frank Davis wrote that: “You should start worrying when bricks start getting thrown through your window or messages daubed on your door." Bauld should emigrate, Davis added, so she would be elsewhere "when your old university department gets torched and your old colleagues are strung up from lamp-posts." Bauld said she had received threatening phone calls while she and a colleague had also had "not particularly pleasant emails" after publishing a review of plain tobacco packaging which led to the government consultation. "A lot of the stuff targeted at me was quite misogynist early on"." (grey lit; Campbell, 2012) | Bhatta, 2020; Mejia, 2008 | Campbell, 2012; Jacobs, 2017; Lorenzo, 2019; McNeil, 2018 |
| Bribery | TOBACCO  India: "A participant was offered undue favours to maintain a distance from TI’s business." (peer-reviewed lit; Goel, 2021)  Nepal: "Advocacy groups were "offered money and other support (whatever they liked) and requested that they remain inactive in the policy making process." (peer-reviewed lit; Bhatta, 2020)  Nepal: "TI offered money to NGOs (especially NCRS [Nepal Cancer Relief Society] and FPCRN [Forum for Protection of Consumer Rights Nepal]) and their lawyer when the cases were pending in the supreme court." (peer-reviewed lit; Bhatta, 2020) | Bhatta, 2020a; Bhatta, 2020b; Goel, 2021 |  |
| Cyberattacks | SSB  Colombia: "[E]mployees at Educar’s offices began to complain about echoes and other voices on their cellphones…Others found it hard to use the internet in the office. In October, Dr Cerón’s office phone ceased functioning entirely. “Sometimes we could not work at all because our laptops would stop following orders and the mouse would just do what it wanted.”" (grey lit; Jacobs, 2017)  Mexico: “Last year, numerous advocates of a proposal to double Mexico’s tax to 20% received strings of upsetting and fraudulent texts from unknown numbers. One man got a message saying his daughter had been seriously injured; another found a text saying his wife was having an affair; a third received a link to a funeral home. Spyware was found on the phones. " (grey lit; Jacobs, 2017)  UNKNOWN SECTOR/COUNTRY  Latin America: "They also infiltrate IT networks and can change the data that you are working on, I've seen it all as well in Latin America..." (grey lit; Mialon, 2021) |  | Jacobs, 2017; MIalon, 2021 |
| Theft/burglary | TOBACCO  Belgium: "Intruders had stolen laptops, petty cash, and bank cards, including: Four laptops from the shared ERS/SFP office, three of which belonged to people working on tobacco control and two laptops from the EPHA office. While desktops and other laptops were not taken, EHPA reported that “both electronic and physical files were targeted including policy and strategy documents, as well as confidential internal documents relating to EPHA’s organisation and staff." (grey lit; TobaccoTactics, 2012) |  | TobaccoTactics, 2012 |
| Other or unspecified threat/intimidation | FOOD  USA: "I wrote the sugar part of that," he said. "When we met in Crete [in June 2000], the sugar people said if the 10% [limit] was in, the whole report would be blocked. I remember we went into a huddle with various people and some of the diplomats, and we were meeting in people's bedrooms and saying, how can we work around this?" In the end, he said, they worked out that a recommendation that nobody should eat sugar more than four times a day was equivalent to a 10% limit. But he considered the committee had been bullied." (grey lit; Boseley, 2003)  USA: "Another ISRF [International Sugar Research Foundation]-sponsored study, by biochemist Walter Pover of the University of Birmingham, in England, had uncovered a possible mechanism to explain how sugar raises triglyceride levels. Pover believed he was on the verge of demonstrating this mechanism “conclusively” and that 18 more weeks of work would nail it down. But instead of providing the funds, the ISRF nixed the project, assessing its value as “nil”." (grey lit; Taubes, 2012)  TOBACCO  Brazil: "Santa Cruz De Suz, it's the tobacco capital in Brazil so all the economy and everything there is around the tobacco industry and the tobacco production...I went there in the discussions on the Framework Convention on Tobacco Control in Brazil and I had to hide in a church like place because if I was seen with some of the people that were like from the farmers movement, the local farmers movement that were interested in conversations about how we could move forward with the health agenda whilst providing safe guards for the small tobacco growers. So, I had to hide because if I was seen together with these people they would probably discredit the movement..." (grey lit; Mialon, 2021)  Europe: "[I]t [The European Bureau for Action on Smoking Prevention (BASP)] evolved as the number on target of the tobacco industry" (peer-reviewed lit; Adamini, 2011)  Finland: "PM [Phillip Morris] had also shown interest in the employees of the tobacco laboratory at the Technical Research Centre of Finland, which monitored maximum limits of harmful substances in tobacco products. Documents contain numerous citations of communications with laboratory employees and descriptions of their character. Trips were organised for them to Neuchâtel in Switzerland, where the European research and development unit of PM (science & technology) was situated." (peer-reviewed lit; Hiilamo, 2003)  India: “Smokeless tobacco lobby always tries to threaten our staff" (An Executive Director of an NGO) (peer-reviewed lit; Goel, 2021)  Thailand: "In another case, TTCs gained powerful international support from the EU, the US, and the UK to pressure the Thai government and tobacco control advocates to extract assurances from the Thai government that officials would not release lists of cigarette ingredients to the public, even though the TTCs had supplied this information to the government." (peer-reviewed lit; Charoenca, 2013)  Ukraine: "Opponents used various strategies during the legislative process to weaken the proposed tax bills, including behind-the-scenes lobbying, use of threats and scare tactics, and formation of front-groups to support the tobacco industry's position." (peer-reviewed lit, Hoe, 2021)  USA: "[A]ggressive activities against the antitobacco programs" AND "a hitlist of proposition 99 funded projects." (peer-reviewed lit; Balbach, 2000)  USA: "Virginia’s ASSIST efforts were hampered by the tobacco industry, which used tactics such as overwhelming the ASSIST 800 number during phone banking…" (peer-reviewed lit; Fallin, 2015)  USA: “[I]n response to proposals by two Massachusetts towns to ban tobacco sales in 1997, tobacco companies encouraged restaurant owners, retailers, smokers and residents to express their opposition at public hearings. In one case, over 50 “partisans and coalition partners” showed up and spoke out against the proposal in the public comment period; in the other, 200–400 reportedly attended a “raucous” public hearing, most of them opposed to the proposal." (peer-reviewed lit; McDaniel, 2020)  UNKNOWN SECTOR/ COUNTRY  Unknown country: *Employer pressure*: "I have another occasion where an employer was pressuring a colleague not to publish with me because the employer wasn't happy with what we were saying - we were denouncing the political practices of the food industry and that was not suiting the employer."(grey lit; Mialon, 2021)  Unknown country*: Suspected research suppression***: “**In my own research for example, I have submitted a paper to a scientific journal, after nine months…they responded that qualitative research wasn't research after all, so they will decline reviewing my article and it took them nine months to do that...the editor was quite close to the industry so I'm not sure, and...he was quite close to the industry in the country I was studying for this specific publication”… (grey lit; Mialon, 2021) | Balbach, 2000; Hiilamo, 2003; Adamini, 2011; Charoenca, 2013; Fallin, 2015; Goel, 2021; Hoe, 2021; McDaniel, 2020; | Boseley, 2003; Taubes, 2012; Mialon, 2021; |
