# Supplementary material for: Intimidation against advocates and researchers in the tobacco, alcohol and ultra-processed food spaces: a review
Source: Health Promot Int. 2024 Nov 21;39(6):daae153. doi: 10.1093/heapro/daae153 (PMC11579607; doi:10.1093/heapro/daae153)
Supplement: daae153_suppl_Supplementary_Files_3 [file daae153_suppl_supplementary_files_3.docx]

Supplementary Table 3. Adjusted responses to intimidation framework

| Responses to intimidation | Explanation |
| --- | --- |
| Carry on as usual | This is about not taking any action in response to threats or attacks and just continuing one’s work as if nothing has happened. |
| Withdrawal | This is about situations in which ongoing projects are abandoned in response to threats or attacks, or when an individual or organisation moves away from an area of work entirely. |
| Defensive adaptation | This is about changes made to projects or ways of working due to intimidation to protect oneself or one’s organisation. |
| Self-censorship | This is about individuals or organisations changing their narratives and, for example, deciding not to speak up publicly. |
| Adapting a project | This is about situations in which projects are amended in response to threats or attacks, for example, changing timeline or focus. |
| Other precautionary measures | This is about actions individuals and organisations take to avoid future intimidation or be better prepared for threats and attacks. This can, for example, be about enhancing IT security, working (more) closely with lawyers and changing social media activity. |
| Offensive action | This is about situation in which individuals or organisations address intimidation in an offensive – rather than defensive – way. |
| Exposing intimidation publicly | This is about making threats or attacks public, for example, by sharing them in the media. |
| Exposing intimidation to relevant stakeholders | This is about sharing experiences of intimidation in a more targeted way. This can be about alerting policymakers or civil servants. |
| Complaints/ legal action | This is about making complaints to the police or other public bodies about the threats or attacks or taking legal action against the aggressor(s). |
| Using intimidation in advocacy work | This is about using experiences of intimidation to inform advocacy work. This can, for example, be about sensitising other advocates in trainings. |
| Correcting misinformation | This is about publicly correcting misinformation presented in the media about the evidence, work or individuals undertaking the work |
